# A Stage of Change Theory–Based, Stage-Matched Intervention for Healthy Dietary Intake Among Office Workers in a Low- to Middle-Income Country: Protocol for a Cluster Randomized Trial

**DOI:** 10.2196/70293

**Published:** 2025-09-30

**Authors:** Janaka Godevithana, Champa Jayalakshmie Wijesinghe, Millawage Supun Dilara Wijesinghe

**Affiliations:** 1 Department of Community Medicine Faculty of Medicine University of Ruhuna Galle Sri Lanka; 2 Centre for Public Health Nutrition Education & Research Faculty of Medicine University of Ruhuna Galle Sri Lanka; 3 Health Promotion Bureau Ministry of Health, Sri Lanka Colombo Sri Lanka

**Keywords:** dietary intake, office workers, stage-matched interventions, stage of change theory, behavior change

## Abstract

**Background:**

An unhealthy diet is a well-established risk factor for the development of noncommunicable diseases, and office workers are at a higher risk of noncommunicable diseases due to their sedentary work style. Stage of change (SOC) theory–based and stage-matched interventions effectively influence dietary and behavior changes. The effectiveness of such interventions in the context of low- and middle-income countries is yet to be assessed.

**Objective:**

This protocol describes a cluster randomized trial planned to evaluate the effectiveness of an intervention for changing dietary behavior among government office workers in the Galle district in Sri Lanka.

**Methods:**

A cluster randomized trial was conducted in 20 clusters divided into intervention and control arms. A cluster was an office with 30 clerical-type workers who were sedentary at work. A stage-matched intervention based on behavior change processes was implemented in the intervention clusters for 3 months. Participants were provided with an intervention matched to their SOC at baseline. Precontemplators and contemplators received awareness-raising and emotional arousal interventions. Others received goal setting and self-monitoring interventions. The SOC and dietary intake were assessed at baseline and the postintervention stage through a staging algorithm, and 24-hour dietary recall was supplemented with a picture guide and computer software. Adherence to the intervention was assessed monthly. We hypothesized that participants would achieve a progressive change in the SOC and healthy dietary intake in the intervention clusters compared to the control clusters.

**Results:**

By December 2024, the planned intervention was completed. Data analysis on the effectiveness of the intervention is to be completed and published in 2025.

**Conclusions:**

This protocol reports a stage-matched intervention based on SOC theory, enriching the current knowledge base with new evidence from office workers in a low- to middle-income country.

**Trial Registration:**

Sri Lanka Clinical Trials Registry SLCTR/2020/025; https://slctr.lk/trials/slctr-2020-025

**International Registered Report Identifier (IRRID):**

DERR1-10.2196/70293

## Introduction

### Background

An unhealthy diet, which can be defined as nutritionally of poor quality or harmful to health [1], has a significant and well-established impact on developing noncommunicable diseases (NCDs) and their complications. It was highlighted that an unhealthy diet accounted for 11 million deaths and 225 million disability-adjusted life years in 2017 [2]. The Food-Based Dietary Guidelines (FBDG) for Sri Lankans provide directions on the necessary amount of food intake and dietary practices for a healthy diet [3]. The FBDG for Sri Lankans have 12 recommendations for a healthy diet, including the amount of food to be consumed [4]. The NCD risk factor survey (STEPS survey) conducted in 2015 highlighted that nearly 75% of male and female individuals were not consuming the recommended amount of fruits and vegetables [5]. An unhealthy diet is common among the workforce, and office workers are at higher risk of developing NCDs due to their sedentary lifestyle at work [6]. Studies conducted in the local context have also highlighted the increased prevalence of unhealthy diets among office workers [7,8]. Because working adults spend most of their time at the workplace, and due to the clustering of common risk factors and behaviors in one place, workplace-based interventions are believed to be successful. Several local and global studies have shown the effectiveness of interventions in workplaces [9,10].

Behavior change does not occur randomly, and behavior change theories explain the mechanism of behavior change [11]. The stage of change (SOC) theory describes 5 stages through which individuals can go through in changing their behavior, and it also describes interventions needed to be conducted at each stage to bring about the behavior change [12]. SOC theory is known to be effective in changing physical activity and dietary behavior, smoking and opioid addiction cessation, and dental hygiene improvement [13], whereas stage-matched interventions have been more successful and effective [14-16].

Behavior change techniques (BCTs) are defined as the smallest active ingredients of an intervention [17]. The selection of appropriate BCTs is one of the key factors on which the effectiveness or the success of an intervention depends [18]. Provision of specific instructions on the desired behavior through a credible source, action planning, goal setting (regarding the behavior or outcome), social support, and self-monitoring are commonly used and effective BCTs for interventions aimed at dietary behavior change. Among them, goal setting and self-monitoring have been identified as effective BCTs in different contexts of behavior change interventions [17,19,20].

### Objectives

Sri Lanka is experiencing a huge burden of NCDs, contributing largely to mortality, morbidity, and health care expenditure [21]. The high prevalence of key risk factors is highlighted in national surveys and regional studies [5]. Office workers are at a higher risk of developing NCDs as the nature of their work leads them to be sedentary during work hours and their dietary intake does not follow local or global recommendations [7,8]. When a group of people with common risk factors are gathered in one place, there is a window of opportunity for health interventions to mitigate these risk factors. Furthermore, health education and promotion interventions through the mainstream preventive health sector hardly ever reach office workers. Therefore, this trial focused on an intervention to change the dietary behavior of office workers in a selected administrative district (Galle) in Sri Lanka. The intervention was planned based on SOC theory and was implemented as a stage-matched intervention at the individual level. Thus, it provided participants with the best-matched intervention. Therefore, this protocol provides valuable insights into several areas, such as dietary interventions for office workers based on behavioral theories and stage-matched interventions. Furthermore, it will enrich the existing scientific knowledge with information on the planning and implementation of interventions in a low- to middle-income country.

This study was designed as a cluster randomized trial with equal allocation of clusters to the intervention and control arms. This protocol was developed to implement an intervention aimed at changing the dietary behavior of office workers.

## Methods

### Design

**Figure 1:**
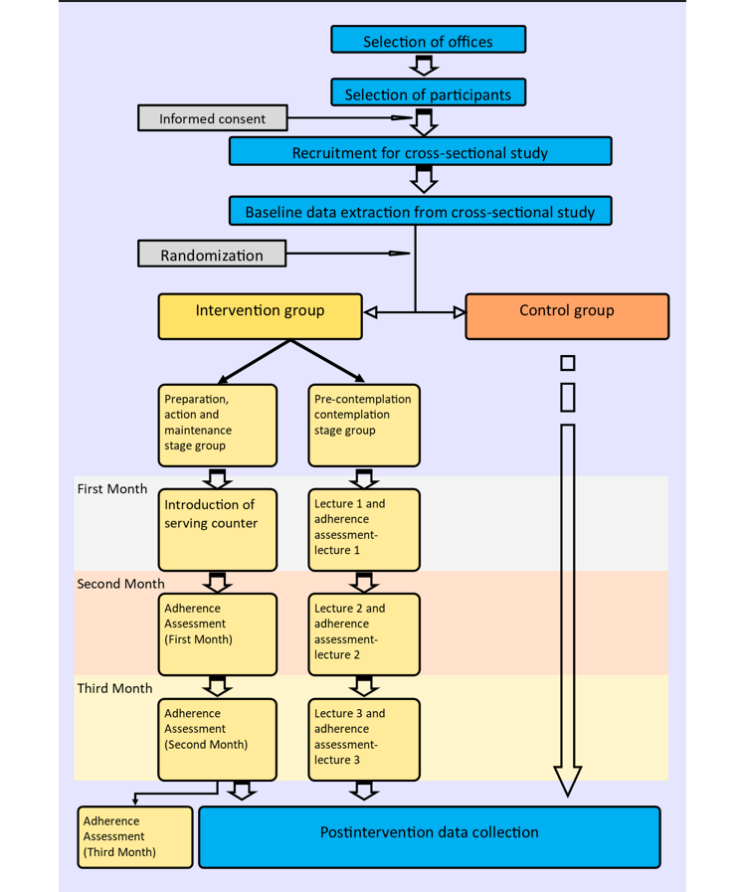
Flow of participants through the study.

This study was a cluster randomized intervention trial with a parallel-group design. It lasted 3 months (12 weeks), and the intervention’s superiority in changing dietary behavior among office workers was assessed compared to the control group. Data for the baseline or preintervention assessment were extracted from a cross-sectional study, which was conducted before the commencement of the intervention to assess the dietary intake of the same study sample, and postintervention data collection was conducted at the end of 3 months. The intervention schedule and enrollment and assessment time points are illustrated in Figure 1.

### Study Setting

This study was conducted in 20 selected government offices in the Galle district. Offices were purposefully chosen to ensure similarity in office functions and the number of clerical-type workers. The minimum travel distance between 2 given offices was more than 5 km. An office with 30 clerical-type workers was defined as a cluster to enroll in the study.

### Study Population and Eligibility Criteria

Clerical workers at government offices selected as clusters were enrolled as study participants. Workers involved in any physical exertion as part of their duties and workers put on specific diet plans for medical reasons were excluded from the study.

### Sample Size

The sample size for the trial was calculated using the following formula for estimating the difference between 2 proportions [22]. A 5% α error was allowed, and 80% power was expected when calculating the sample size. A 50% increase in the factor of interest was assumed, as a meta-analysis on behavioral modifications showed an increase in factors of interest by 50%. As the 2015 STEPS survey reported that only 28% of the population had met the recommended number of fruit and vegetable servings [5], 0.3 was assumed as the baseline proportion for the recommended fruit and vegetable intake and was expected to increase by 50% at the end of the intervention. Therefore, 0.45 was assumed as the proportion in the intervention arm after the intervention.

Substituting the aforementioned values in the equation, the calculated minimum sample size in each arm of the cluster randomized trial was 160.

Design effect for cluster sampling was calculated assuming a cluster size of 30 and an intraclass correlation of 0.02 because the intraclass correlation coefficient in human studies usually lies between 0.01 and 0.02. A study among office workers reported an intraclass correlation coefficient of 0.02 [23]. Substituting the aforementioned values in the equation, the design effect for this study was 1.58.

Therefore, the minimum sample size for each arm was 253. After accounting for a 10% dropout rate, the study’s minimum sample size per arm was 280 office workers. Thus, the minimum sample size in the intervention and control groups was 560 office workers.

### Recruitment

Multistage cluster sampling was used to recruit study participants. A cluster was defined as an office with at least 30 clerical-type workers. A total of 20 clusters were selected purposefully to fulfill three requirements as follows: (1) the nature of the work carried out in the offices should be similar, (2) travel distance between any selected offices should be 5 km, and (3) the offices should have 30 to 40 clerical-type workers.

An exhaustive list of government offices established in the Galle district was prepared after obtaining information from the chief secretary’s office (Southern Province) and the District Secretariat (Galle district). Information about the functions of each office was obtained from the district secretary and the chief secretary’s office. Offices that carried out similar activities were short-listed. Offices were then short-listed again depending on the number of clerical-type workers (30 to 40). The travel distance between each office was then assessed using the direction function in Google Maps. If the travel distance between 2 or more offices was less than 5 km, the office with the highest number of clerical-type workers was selected, excluding the rest. Short-listing aimed to select 20 offices that matched the recruitment criteria. However, the ultimate short list contained only 19 offices because of inadequate distance between 2 offices. The distance requirement was then reduced to at least 5 km from more than 5 km.

Within the clusters, participants were selected using a series of random numbers. Once clusters were selected, the relevant offices were contacted, and a study coordinator was appointed for each office. The coordinator obtained a list of clerical-type workers’ names. The workers’ names were arranged alphabetically, and a number was assigned to each. An web-based random number generator selected 33 numbers from the list. The workers assigned the selected numbers were invited to participate after assessing their eligibility according to the inclusion and exclusion criteria. They were informed of the study and the data collection date through the office coordinator.

On the day of data collection, the selected participants were informed about the study, and the purpose, risks, and benefits were explained. The workers who provided consent were recruited for the study

### Participant Timeline

Data from a cross-sectional study were extracted for all selected government offices in the Galle district, and 2 months were allocated for this. Data on participants’ demographic profiles, health conditions, dietary practices, SOC, and dietary intake were extracted from the aforementioned cross-sectional study as preintervention or baseline data for the interventional study. Randomization was conducted following the baseline data collection. Once randomization was done, the principal investigator and resource person for lectures visited each office in the intervention arm on a prescheduled day. All participants in the study were informed of the date beforehand. The principal investigator applied the intervention, and the resource person delivered lectures. If any participant missed the initial intervention, a second visit was arranged to achieve maximum participation. The initiation of the intervention in all 10 selected offices was completed within 1 month. Monthly assessments and lectures were conducted on prescheduled days following one, two and three months of the initiation date for each office. However, a second day was not arranged for monthly assessments and lectures. A postintervention assessment was conducted at the end of 3 months. The date for the postintervention evaluation was given to all participants to ensure that they took part in the assessment. If any participant missed the postintervention assessment, another day was arranged. Figure 1 shows the flow of participants through the intervention process.

### Data Collection

After selecting offices for the study, administrative approval was obtained from relevant authorities. The principal investigator met or contacted the office heads (divisional secretaries and chairmen of local authorities) to obtain permission for data collection. Thereafter, one senior worker, either a development officer or management assistant, was appointed as the coordinator for the study from each office. The list of eligible office workers was obtained through the coordinator. Thereafter, 33 workers were selected from each office as described in the Recruitment section. A date for data collection was fixed, and all selected participants were informed through the coordinator, requesting their presence at the office on the fixed date.

The principal investigator explained the purpose and nature of the study to potential participants. Information sheets and consent forms (Multimedia Appendix 1) were distributed among all eligible participants invited to take part in the study. The self-administered questionnaire (Multimedia Appendix 2) was then distributed among participants who consented to the study.

Three trained data collectors collected the data. Participants were allowed to ask questions and supported by the data collectors. After completion of data collection, the data collectors retrieved the filled-out questionnaires and checked them for completeness.

Furthermore, participant-reported health-related data were verified by referring to available clinical records as participants were asked to keep clinical records with them before data collection.

The data collectors also measured the weight and height of the participants using a calibrated weighing scale and stadiometer, and the measurements were recorded following the standard procedure described by Greenwood et al [24].

The assessment of the SOC was conducted using a staging algorithm which was originally used in the well-known “5 a day” project and subsequently used by many studies [25-27]. It comprised 4 mutually exclusive branching questions . The responses to the 4 questions were used to determine a participant’s SOC as follows.

The first question was “Do you follow healthy dietary guidelines?” If the answer was “yes,” the participant was asked, “for how long have you practiced your current diet?” (response options: “more than 6 months” [maintenance stage] and “less than 6 months” [action stage]). If the answer was “no,” the participant was asked, “are you seriously thinking of changing to a healthy diet in the next 6 months?” If the answer to the latter was “yes,” the participant was asked, “are you going to change your dietary habits next month?” If the answer was “yes,” the participant was allocated to the preparation stage. If the answer was “no,” the participant was allocated to the contemplation stage. If the answer to the question “are you seriously thinking of changing to a healthy diet in the next 6 months?” was “no,” the participant was allocated to the precontemplation stage.

To minimize bias due to misperception of the diet’s healthiness, the initial question was modified to read the following: “Have you changed your diet by reducing the amount of carbohydrates, salt, and sugar and increasing the amount of fruit and vegetable portions?”

The SOC of a participant was determined by coupling the staging algorithm with dietary assessment as suggested by Greene et al [28]. The perceived SOC reported by the participants was amalgamated with their dietary intake. Participants who followed a diet that met the definition of a healthy diet were assigned to their perceived SOC as reported by them. For example, if a participant reported being in the action or maintenance stages and their diet met the overall definition of a healthy diet, they were allocated to their reported SOC. In contrast, if participants who followed a diet that did not meet the definition of healthy dietary intake reported being in the action or maintenance stage, they were allocated to the preparation stage. This approach is summarized in Table 1.

**Table 1:** Summary of the process of ascertaining the stage of change of the participants.

Staging algorithm result	Type of diet based on 24-h recall	
	Healthy	Unhealthy
Precontemplation	Precontemplation	Precontemplation
Contemplation	Contemplation	Contemplation
Preparation	Preparation	Preparation
Action	Action	Preparation
Maintenance	Maintenance	Preparation

The 24-hour dietary recall was completed for each participant by the trained data collectors through face-to-face interviews and a computer software designed specifically for this study. Participants were asked to recall their food intake the day before the data collection date. Data were collected on a routine office day. Mondays and days after holidays were not scheduled for data collection. At the same time, it was established that participants would have their regular meals on the date selected for dietary recall. Where this was not possible, the nearest day in which they had their regular meals as they did on a routine office day was considered for the dietary recall.

Participants were asked to name all food items and the amounts they consumed between waking up and going to sleep. Data collectors then inquired about the amount of food consumed using household measurements.

Interview materials were used to quantify the intake from different food groups. A picture guide that included all household measuring items (eg, spoons and cups) and food portions obtained through commonly used measurements was used to identify the quantity of each food item consumed (Multimedia Appendix 3). A computer software that can convert household measurements to servings described in the FBGD for Sri Lankans was used to calculate the serving number from each food group (Multimedia Appendix 4).

### Randomization

After baseline data collection, all selected clusters were randomized into 2 arms. The principal investigator or data collectors did not know whether a particular cluster belonged to the control or intervention arm at baseline data collection, which minimized observer bias during this stage.

All selected clusters were arranged in ascending alphabetical order, and a number was assigned to each cluster. An online randomization sequence generator assigned the numbers 1 to 20 into 2 arms. The intervention arm had 10 numbers, and the control arm had 10 numbers. Clusters with allocated numbers were randomly assigned to the intervention and control arms.

### Allocation Concealment

The principal investigator enrolled the clusters in the study. The randomization sequence generation was delayed until baseline data collection was completed, ensuring that the principal investigator who enrolled the clusters was unaware of the randomization sequence at the time of enrollment.

The randomization sequence was generated by an independent researcher (not a research team member) from a different district. He was asked to allocate numbers from 1 to 20 into the intervention and control groups using a random sequence generator. He was unaware of the cluster list and the allocated number for each cluster. He did not know about the offices and their distribution in the Galle district as he was selected from a different district. This independent sequence generation randomized clusters into the intervention and control arms without manipulation.

### Intervention

#### Overview

The intervention was developed to match the SOC of the participants.

It has been highlighted that different SOCs require different processes of change to progress from one stage to another. Prochaska and Velicer [12] have described the relationship between SOCs and the processes of change that correspond to each one (Textbox 1).

**Stages of change and the processes of change that correspond to each one.**

**Precontemplation**

- Consciousness raising
- Dramatic relief
- Environmental re-evaluation

**Contemplation**

- Consciousness raising
- Dramatic relief
- Environmental re-evaluation
- Self–re-evaluation

**Preparation**

- Self- and social liberation

**Action**

- Contingency management
- Helping relationship
- Counterconditioning
- Stimulus control

**Maintenance**

- Contingency management
- Helping relationship
- Counterconditioning
- Stimulus control

According to Prochaska and Velicer [12], people in the precontemplation and contemplation stages need more consciousness raising, dramatic relief, self–re-evaluation, and environment re-evaluation. In these processes, they are provided with information on healthy and unhealthy behavior. Furthermore, the emotional and imaginary comparison of healthy and unhealthy behavior is facilitated. Similarly, the later stages, such as preparation, action, and maintenance, need processes of change such as self-liberation, contingency management, helping relationships, counterconditioning, and stimulus control. In these processes, more emphasis is placed on replacing the unhealthy behavior with a healthy one, monitoring whether the participant is on the correct path, and ensuring support from the environment [12].

Therefore, interventions to raise awareness and provide emotional arousal were designed for the precontemplation and contemplation stages. In contrast, interventions supporting and monitoring healthy behavior were designed for the preparation, action, and maintenance stages.

For precontemplators and contemplators, a 30-minute awareness-raising lecture was conducted at the beginning of each month during the intervention period of 3 months, and the following topics (one topic for each month) were discussed in each lecture in the order given (Multimedia Appendices 5-7): NCDs and their risk factors; risks of an unhealthy diet and benefits of a healthy diet, as well as strengths, weaknesses, opportunities, and threats analysis for behavior change; and how to change to a healthy diet.

While providing information to increase awareness, emotional arousal and self–re-evaluation were incorporated into all 3 lectures. The benefits of a healthy diet were highlighted, and participants were encouraged to contemplate the benefits of a healthy diet and the harmful effects of an unhealthy diet.

The lectures were delivered by a medical officer who was competent in public speaking and experienced in NCD prevention activities. The content of the lectures was discussed with the lecturer, and the objectives and methodology of the study were explained. It was emphasized to the lecturer to ensure similarity in the presentation and points highlighted in the lectures delivered to all intervention clusters. A slide show was prepared, and the lecturer and principal investigator agreed on the points to be discussed with each slide. A narrated lecture was circulated (through email or other electronic media) to the participants who were not able to take part in the lecture.

A separate intervention was designed for the preparation, action, and maintenance stages aiming to support and monitor the healthy behavior to be started or already started.

The intervention introduced a daily serving counter designed by the principal investigator based on serving recommendations published in the FBDG for Sri Lankans [4]. It allowed the participants to record their daily intake of servings of different food groups. The recommended servings were indicated for each food group. The lower and upper limits of recommended servings, where they are considered unhealthy, were indicated in red. A guide on serving sizes for each food group was added (Multimedia Appendix 8).

In addition, a mobile app with similar functions (the serving guide and the ability to record servings) was also developed for smartphone users.

#### Assessment of Adherence to the Intervention

Attendance at the in-person lectures was recorded. Feedback was obtained from those who were provided with the narrated lectures, asking them whether they watched the presentation. If no feedback was received within 5 days of receiving the narrated lecture, it was considered as nonparticipation in the lecture.

The principal investigator reviewed the marking of the serving counter monthly, visiting the office at the end of each month. Participants were informed of the date of the investigator’s visit and asked to bring the marked serving counters to be checked for completeness. If a participant missed more than 2 marking days per week during the month, it was considered poor adherence to the serving counter.

Participants were advised to attend the lectures and serving counter assessment sessions. After a discussion with participants, the dates and times for lectures and investigator visits were arranged to ensure maximum participation.

### Control

No intervention was implemented for the control clusters. However, they were asked not to refrain from attending any routine health education or promotion activities conducted by other health care institutions and persons.

### Outcome Measures

#### Overview

Postintervention assessment was conducted at the end of the intervention period in the same manner for the intervention and control clusters. The same data collectors who conducted the baseline (preintervention) data collection were recruited for the postintervention data collection. However, the study arm (intervention or control) to which an office belonged was not disclosed to the data collectors.

SOC data were collected using the same staging algorithm as in the baseline or preintervention data collection. The same 24-hour dietary recall with the picture guide and computer software were used to collect data on dietary intake at the postintervention assessment.

#### Primary Outcome

The primary outcome of the intervention was a progressive change in the SOC, which was defined as *progression from a lower SOC to a higher SOC*.

#### Secondary Outcome

The secondary outcome was a change from an unhealthy to a healthy diet or persistence in a healthy diet.

A healthy diet was defined as adherence to the recommended servings in the FBDG for Sri Lankans for more than 3 food groups, including cereal and cereal-based foods, fruits, and vegetables, with the consumption of 1 or no unhealthy foods per day. The recommended servings of different food groups per day are shown in Table 2.

**Table 2:** Recommended number of servings of different food groups per day.

Food group	Number of servings
Rice, bread, other cereals, and yams	6-7 (6-11^a^)
Fruits	2-3
Vegetables	3-5
Milk or milk products	1-2
Fish, pulses (legumes), meat, and eggs	3-4
Nuts and oily seed	2-4

^a^Wider allowance was given to the rice, bread, other cereals, and yams food group in the Food-Based Dietary Guidelines as the energy requirements differ with activity level and sex. Because office workers were considered a sedentary group based on the nature of their duties, 6 to 7 servings of the rice, bread, other cereals, and yams food group were considered the recommended number of servings based on expert opinion.

The FBDG recommend variety in the food items consumed from each food group and in the number of servings specified in Table 2. However, the routine Sri Lankan dietary intake does not include all 6 food groups in daily meals. Therefore, we considered the 3 most consumed and important food groups (cereals, vegetables, and fruits) to be included in the definition of a healthy diet. In addition, we included a limit for unhealthy foods (foods containing only sugar and fat and highly processed foods) in the definition.

### Data Analysis

The distribution of basic sociodemographic characteristics, NCD status, dietary behaviors and practices, and work-related characteristics of the participants in the intervention and control arms will be assessed to determine whether the 2 groups were comparable at baseline.

Bivariate and multivariate analyses will be used to assess the effectiveness of the intervention. Progressive improvement in the SOC will be used as the primary outcome. Those who underwent a progressive change in their SOC will be coded as 1, and those who did not undergo such a change will be coded as 0. Similarly, those who changed from an unhealthy to a healthy diet or persisted in a healthy diet will be coded as 1, and those who did not will be coded as 0. Furthermore, changes in the intake of individual food groups to recommended levels will be coded as 1 and 0 similarly.

Bivariate analysis will use the primary and secondary outcomes as the dependent variables and sociodemographic factors, NCD status, dietary behaviors, and social relations as the independent variables. The chi-square test, independent-sample *t* test (2-tailed), and Mann-Whitney *U* test will be used as appropriate. Multivariate analysis will be conducted using binary logistic regression with the forward selection option to build the model. The variables included in the model will be determined based on demonstrating significant associations with the outcome variables in the bivariate analysis.

Sociodemographic factors, NCD status, dietary behaviors, social relations, and the allocated arm of the participants lost to follow-up will be compared with those of other participants to ensure no selective data loss. Further analysis will be conducted separately under two assumptions as follows: (1) all participants lost to follow-up achieved the primary and secondary outcomes, and (2) all participants lost to follow-up did not achieve the primary and secondary outcomes.

Odds ratios will be reported for the 2 assumptions separately. The level of probability will be set at .05 for all analyses.

### Ethical Considerations

Ethical clearance for this study was obtained from the Ethical Review Committee, Faculty of Medicine, University of Ruhuna (registration 2020/P/105). This study was registered in the Sri Lanka Clinical Trials Registry. Administrative approval was obtained from relevant authorities at the provincial and district levels. Furthermore, this study obtained the approval of the Regional Director of Health Services of the Galle district. This study was conducted while adhering to the World Medical Association Declaration of Helsinki on ethical principles for medical research involving human participants.

Written informed consent was obtained from all study participants. They received a comprehensive information sheet and a consent form, which each participant had to complete and sign. Consent was 100% voluntary through being assured that declining to participate in the study would not affect the way in which they were treated at the office or a health care facility.

Participants were allowed to refuse to take part in all or part of the study or refrain from doing any activity requested by the researchers. They were given the freedom to withdraw from the study at any time without any effect on their routine work and personal life. They were assured that there would not be any difference in treatment toward those who refused to participate or withdrew from the study.

Two main risks for the participants were identified. First, there could be breaches of privacy when sharing personal information with researchers. Second, participants could be subjected to a behavior change if they were allocated to the intervention group. Identified workers with problems were referred to the nearest health care facility with their consent.

Although participants shared their personal information, those data are being kept anonymous and not shared with anybody other than the research team. Confidentiality is being ensured by keeping the data under lock and key and in password-protected computers. A serial number is being used to identify the participants during data entry and storage. When disseminating data, the names or identification details will not be included.

The participants were advised to take part in the awareness program and change their unhealthy dietary behaviors. These evidence-based activities were included in national and international guidelines on healthy lifestyles, so they have been proven to be more beneficial than harmful to participants. Therefore, no compensation was provided for participants as the intervention did not harm them, and they were asked to make minor changes in their lifestyle.

The findings of this study are planned to be disseminated only in scientific reports, publications, and forums. It has been ensured that no identifiable personal information will be shared in such dissemination activities. The scientific community and public can access these publications and use them for the benefit of mankind.

## Results

**Figure 2:**
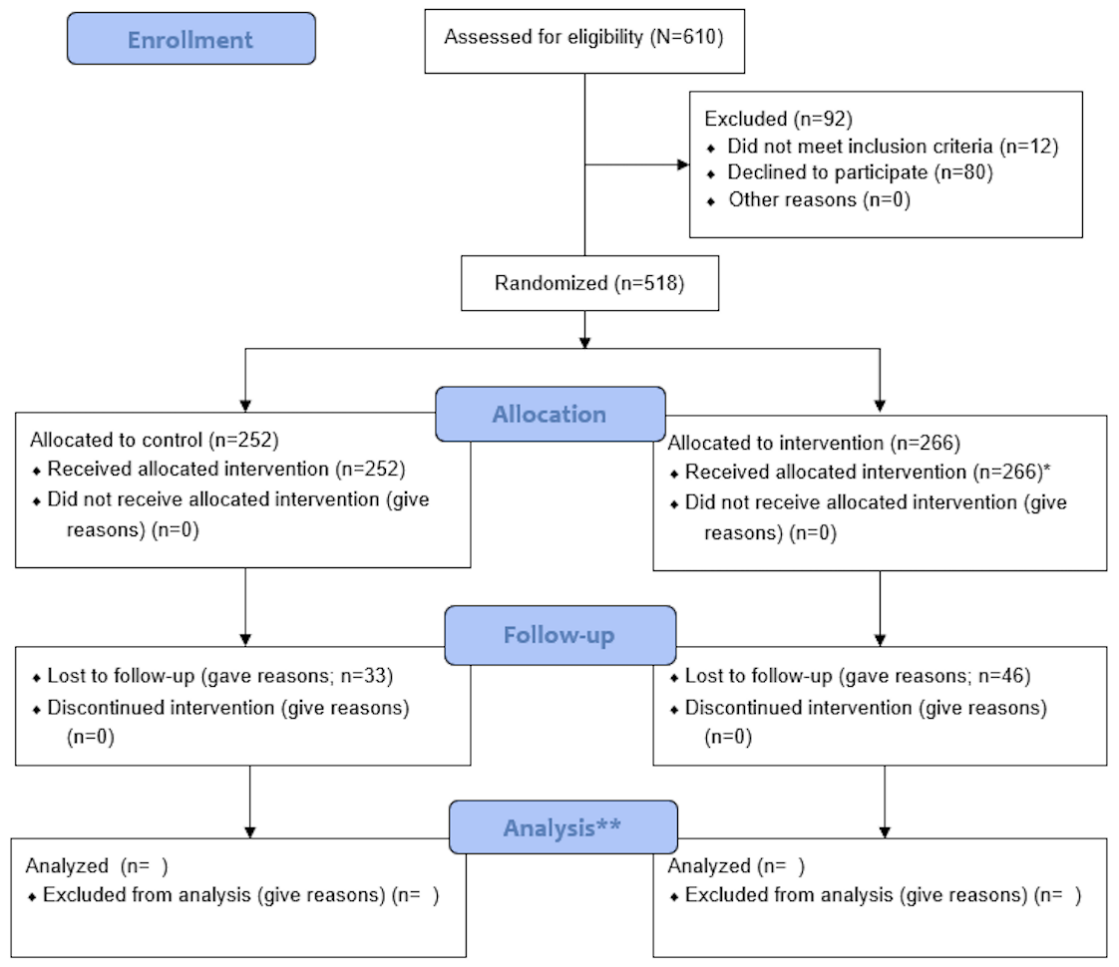
CONSORT (Consolidated Standards of Reporting Trials) flow diagram for a stage of change theory–based, stage-matched intervention for healthy dietary intake among office workers. *The intervention group was divided into 2 groups depending on the participants’ stage of change at the beginning. Two different interventions were implemented for each group. One group included 143 participants at the beginning and 127 participants at the end, and the other included 123 participants at the beginning and 97 participants at the end. **Postintervention analysis has not yet been completed.

As of December 2024, baseline data collection, participant enrollment, intervention administration, and postintervention data collection have been completed. Altogether, 610 workers from 20 offices were screened for eligibility. Of the 610 workers, 92 (15.1%) were excluded due to not meeting the eligibility criteria (n=12, 13%) and not consenting (n=80, 87%). A total of 518 participants were recruited for the baseline assessment, and then 20 offices were randomized to the control and intervention groups. The intervention group comprised 51.4% (266/518) of the participants, whereas the control group comprised 48.6% (252/518) of the participants. Ultimately, 439 participants (n=220, 50.1% in the intervention group and n=219, 49.9% in the control group) were included in the postintervention data collection. Figure 2 shows the CONSORT (Consolidated Standards of Reporting Trials) flowchart up to the completion of follow-up. Data analysis of the effectiveness of the intervention is to be completed and published. Data collected at baseline (before the intervention) were published as a descriptive study on dietary intake among office workers. However, none of the data related to the intervention have been published.

## Discussion

This study assessed an intervention’s effectiveness in making office workers’ diets healthier. Because dietary behaviors are predominantly specific for individuals, interventions to change such behaviors should match the individuals’ characteristics. Furthermore, it has been proven that interventions based on behavioral theories are more successful and more straightforward to assess and evaluate [29]. This protocol describes a stage-matched intervention using the SOC theory. Nutritional interventions based on SOC were found to be effective in recent studies and reviews [13,29,30]. Findings of different systematic reviews and interventional studies emphasize that stage-matched interventions have been more successful and effective in achieving the desired change in behavior [14-16]. However, no dietary behavior change interventions of this nature (SOC based and stage matched) for office workers were found in the available literature. Therefore, this trial will fill the knowledge gap on SOC-based and stage-matched interventions for office workers.

Furthermore, this study will enrich the existing research evidence with findings from a low- to middle-income country in the Southeast Asian region. In addition, it will help local-level policymakers in planning interventions to uplift the health of this specific group of people who are at a higher risk of NCDs due to their sedentary working conditions.

However, this study’s major limitation is the inability to generalize the results. Because only clerical-type workers were included in this study, the results cannot be generalized to all office workers. Randomization at the cluster level does not fully eliminate the influence of other factors, which may affect the effectiveness of the intervention and, ultimately, the study’s overall results. Therefore, this study’s analysis and results must be interpreted with caution.

Ultimately, this study is expected to bridge the knowledge gap on dietary interventions for office workers, and the limitations must be considered when assessing the findings.

## Appendix

Multimedia Appendix 1 Information sheet.

Multimedia Appendix 2 Questionnaire.

Multimedia Appendix 3 Picture guide for dietary recall.

Multimedia Appendix 4 Computer software to calculate food servings.

Multimedia Appendix 5 Lecture 01 for precontemplators and contemplators.

Multimedia Appendix 6 Lecture 02 for precontemplators and contemplators.

Multimedia Appendix 7 Lecture 03 for precontemplators and contemplators.

Multimedia Appendix 8 Guide on serving sizes for each food group.

## Abbreviations

BCT: behavior change technique
CONSORT: Consolidated Standards of Reporting Trials
FBDG: Food-Based Dietary Guidelines
NCD: noncommunicable disease
SOC: stage of change
STEPS: noncommunicable disease risk factor survey

## Data Availability

Data sharing is not applicable to this paper as no data sets were generated or analyzed during this study.

All the relevant items to this study are included in this published article (and its multimedia appendices).
