# Supplementary material for: A Stage of Change Theory–Based, Stage-Matched Intervention for Healthy Dietary Intake Among Office Workers in a Low- to Middle-Income Country: Protocol for a Cluster Randomized Trial
Source: JMIR Res Protoc. 2025 Sep 30;14:e70293. doi: 10.2196/70293 (PMC12521855; doi:10.2196/70293)
Supplement: Multimedia Appendix 1 [file resprot_v14i1e70293_app1.docx]

This informed consent form is for government office workers in Galle district and who we are inviting to participate in phase I of research titled “Intervention to change for a healthy diet within office community of government offices in Galle district”

**This Informed Consent Form has two parts:**

• **Information Sheet (to share information about the study with you)**

• **Certificate of Consent (for signatures if you choose to participate)**

**You will be given a copy of the full Informed Consent Form**

**Part I: Information Sheet**

**Introduction**

I am, Dr G. J. Chandana, conducting a research study on Intervention to change for a healthy diet within office community of government offices in Galle district research project is supervised by Dr C. J. Wijesinghe, senior lecturer at department of community medicine, University of Ruhuna.

I am going to give you information and invite you to be part of this research. You must decide now if you want to participate in this research. Before you decide, you can talk to anyone you feel comfortable with about the research. This consent form may contain words that you do not understand. Please ask me to stop as we go through the information and I will take time to explain. If you have questions later, you can ask them of me.

**Purpose of the research**

Non-Communicable Diseases (NCD) have been major problem in global and local context in recent past. Smoking, harmful use of alcohol, unhealthy diet and sedentary lifestyle were found to be as major risk factors for NCDs. It has been pointed out in many instances that office workers are more prone for unhealthy diet and sedentary lifestyle due to their nature of work.

This study will assess dietary practices of office workers and will devise an intervention to achieve healthy dietary practices and assess its effectiveness. This study will assess dietary practices of office workers. At the same time effectiveness of an intervention targeted on behavior change in office workers will be assessed during the study.

**Type of Research Intervention**

During this study your office will be considered as a cluster (a place with boundaries where a similar kind of people gathered together). Such clusters selected for the study will be further divided in to two groups using random sequence. Two groups will be control and intervention group. At the beginning of the study data regarding your dietary practices and stage of change for a behavior change. This data will be collected as follows;

You will be provided with the questionnaire and you will be asked 20 questions on usual socio-economic data, general health condition and food consumption related data. Further you will be asked another four questions about your intention to change for a healthy diet. Then you will be asked to recall dietary intake of food where you will be questioned on food intake within 24 hours of the previous date by a data collector who is a medically qualified. You will be asked on both meals and snacks and amount you have taken. Data collectors will assist you to quantify the amount you had with visual scale of sizes of spoons, cups, food portions etc.

Following this data collection you will be getting the intervention for behavioral change depending on the group (control/intervention) to which your office is categorized into. If your office is categorized to intervention arm depending on your stage of change you will be getting following interventions

If you haven’t thought of changing your dietary behavior you will be getting 30 minutes lecture at the beginning of every month for three month period. And you will be reminded on content covered in lectures through text messages or short video clips shared with you through social media.

If you are planning to change your diet in to a healthy diet or already changed to a healthy diet you will be provided with a serving counter which indicate the amount of food you can have per a day and you have to tick number of servings you have for a day. Serving counter will be either a smart phone app which is made for the purpose or a card which contain number of servings as check boxes to tick.

If your office is categorized to control arm all of your office co-workers will receive a one hour lecture on healthy diet for prevention of NCD.

Intervention period will last for three months duration and monthly assessment on adherence to the intervention will be done by chief investigator or a data collector.

At the end of three month period you will be again asked for 24 hour dietary recall and stage of change in relation to having healthy diet.

**Participant Selection**

You are being invited to take part in the study because you are a government office worker your office have been selected for the study.

**Voluntary Participation**

The choice that you make will have no bearing on the healthcare services you receive.

**Procedures**

All workers at your office will be selected for obtaining data for the research project and will be invited to take part in the study. If you are willing to participate, you will be provided with the self-administered part of the questionnaire which contain 24 questions and may take 15 to 20 minutes to complete. If you feel it is difficult to fill it you can ask for help from me or a data collector.

I and few data collectors will visit your office on a convenient date for you to pose minimum disturbance to your routine works. We will be asking you regarding food that you have consumed within 24 hours duration of the previous day. We will be asking you about all main meals and snacks you had. You will be asked regarding food type and amount you had. You will be shown photographs of different sizes of cups and spoons and food portion measured by that particular food. You will have to specify the amount of food you had using such photograph. Data on types and amount of consume food will then and there entered in to a computer software to calculate number of food serving that defined in food based dietary guidelines which recommends the amount of food that an from each type and amount of food to consume to be healthy.

Following this data collection you will be receiving intervention or control group activities as described in the section on type of research intervention. The intervention period excluding data collection period will last for three months duration and serial monthly assessments will be carried out for adherence to intervention activities. At the end of three month intervention period you will be again asked for 24 hour dietary recall and stage of change in relation to having healthy diet

**Risks**

There is a risk that you may share some personal or confidential information by chance, or that you may feel uncomfortable talking about some of the topics. However, we do not wish for this to happen. If you change your mind, and no longer wish to participate, just say so. We can stop at any time.

During the intervention you may have to change your routine behavior which you have been used to be and it might be difficult to you. But your behavior change or the new behavior is the one recommended in the food based dietary guideline for Sri Lanka.

**Benefits**

If you are selected to intervention group you will be provided with an intervention that is tailored to your stage of change regarding healthy dietary practices and your body mass index (BMI). So you will be facilitated to achieve healthy dietary practices and hence your risk of having NCD will be reduced.

Though you have been selected to control group still you will be receiving a one hour lecture on healthy diet for prevention of NCD

**Confidentiality**

We will not share information about you to anyone outside of the research team. The information that we collect from this research project will be kept private. Any information about you will have a number on it instead of your name. You will be given a number when you selected for the study. All your data will be recorded under that serial number. Only the researchers will know what your number is and we will lock that information up with a lock and key. It will not be shared with or given to anyone.

**Sharing the Results**

Nothing that you tell us will be shared with anybody outside the research team, and nothing will be attributed to you by name. In a few months, we will publish the overall results of our research so that both you and other interested people may learn from the research.

**Right to Refuse or Withdraw**

You do not have to take part in this research if you do not wish to do so, and choosing to participate will not affect your job or job-related evaluations in any way. You may stop participating in the interview at any time that you wish without your job being affected. If you choose to withdraw the research, any audio or paper data will be immediately destroyed.

**Who to Contact**

If you have any questions, you can ask them now or later. If you wish to ask questions later, you may contact me Dr. G.J.Chandana, 0779040090, janakagode@gmail.com.

This proposal has been reviewed and approved by Ethics Review Committee at faculty of medicine of University of Ruhuna, which is a committee whose task it is to make sure that research participants are protected from harm and proposed study will not violate ethical principle during planning, implementation and publishing stages. If you wish to find about more about the IRB, contact 091 2232801. You can also ask me any more questions about any part of the research study, if you wish to. Do you have any questions?

**Part II: Certificate of Consent**

I have been invited to participate in research on dietary practices among office workers.

I have read the foregoing information, or it has been read to me. I have had the opportunity to ask questions about it and any questions I have been asked have been answered to my satisfaction. I consent voluntarily to be a participant in this study

**Name of Participant__________________**

**Signature of Participant ___________________**

**Date ___________________________**

**Day/month/year**

**Statement by the researcher/person taking consent**

I have accurately read out the information sheet to the potential participant, and to the best of my ability made sure that the participant understands the nature and procedure of data collection.

I confirm that the participant was given an opportunity to ask questions about the study, and all the questions asked by the participant have been answered correctly and to the best of my ability. I confirm that the individual has not been coerced into giving consent, and the consent has been given freely and voluntarily.

**A copy of this ICF has been provided to the participant.**

**Name of Researcher/person taking the consent________________________**

**Signature of Researcher /person taking the consent__________________________**

**Date ___________________________**

Day/month/year

To whom it may concern

Dear Sir/Madam/collogue,

**Regarding Mr. G.V. Sunil**

I, Dr. G.J. Chandana (SLMC 31635), have been the family doctor for Mr. G.V.Sunil for more than one year duration and he has been suffering from **type II diabetes** **mellitus** for 12 years and **hypertension** and **dyslipidemia** for two years duration. Further he is suffering from whole spectrum of diabetes complication including vasculopathy, retinopathy and neuropathy. He has had recent history of **minor stroke** resulting disability in right hand.

With above mention conditions he is having difficulties in performing routine activities which he used to do

I kindly request you to consider those in any decision going to be taken on him by you.

Thank you

Yours faithfully

…………………….

Dr. G.J. Chandana (MBBS, MSc com. Medicine)

Medical Officer

SLMC 31635
