# Supplementary material for: A Stage of Change Theory–Based, Stage-Matched Intervention for Healthy Dietary Intake Among Office Workers in a Low- to Middle-Income Country: Protocol for a Cluster Randomized Trial
Source: JMIR Res Protoc. 2025 Sep 30;14:e70293. doi: 10.2196/70293 (PMC12521855; doi:10.2196/70293)
Supplement: Multimedia Appendix 2 [file resprot_v14i1e70293_app2.docx]

**Annexure IV**

**Questionnaire for the study on dietary practices, social relations and intervention to change for a healthy diet within office community of government offices in Galle district**

| **Office name** | **Office category** | **Serial number** |
| --- | --- | --- |
|  |  |  |

**Part 01 - Socio-demographic data**

1. Age at last birthday:

2. Sex:

| Male |  | Female |  |
| --- | --- | --- | --- |

3. Residential area (GA Division)…………………………

4. Ethnicity:

| Sinhala |  |
| --- | --- |
| Tamil |  |
| Muslim |  |
| Burgher |  |
| Other |  |

5. Religion: -

| Buddhist |  |
| --- | --- |
| Hindu |  |
| Islam |  |
| Christian |  |
| Other |  |

6. Highest level of education attained: -

| No school education |  | Passed G.C.E.(Advanced level) |  |
| --- | --- | --- | --- |
| Grade 1-5 |  | Tertiary education (diploma/ degree or equivalent) |  |
| Grade 6-11 |  |  |  |
| Passed general certificate of education(G.C.E) (ordinary level) |  |  |  |

7. Marital status:

| Unmarried |  |
| --- | --- |
| Living together |  |
| Married |  |
| Married and separated |  |
| Divorced |  |
| Widowed |  |

8. Present post:

| Managerial |  |
| --- | --- |
| Professionals |  |
| Technical and associate officers |  |
| Clerical and supportive worker |  |

9. If married, spouse’s occupation:

| Managerial |  |
| --- | --- |
| Professionals |  |
| Technical and associate officers |  |
| Clerical and supportive worker |  |

10. If the spouse is employed, do both work at same station?

| Yes |  | No |  |
| --- | --- | --- | --- |

11. Number of children: …………………………….

12. Average monthly family income:

13. Distance to workplace form home / routine place of stay: (time taken to come to office by using routine method of transport)

| Less than 30 minutes |  |
| --- | --- |
| 30 minutes – 1hour |  |
| More than 1 hour |  |

14. Mode of traveling:

| Walking |  |
| --- | --- |
| Public transport |  |
| Private vehicle (own/shared) |  |

**Part 02 - Health status**

15. Presence of any long term illnesses:

| Diabetes |  |
| --- | --- |
| Hypertension |  |
| Dyslipidaemia |  |
| Ischaemic Heart Disease |  |
| Stroke |  |
| Other (specify) |  |

16. Family history of Non Communicable Diseases:

| Diabetes |  |
| --- | --- |
| Hypertension |  |
| Dyslipidaemia |  |
| Ischaemic Heart Disease |  |
| Stroke |  |
| Other (specify) |  |

**Part 03 - Food consumption related data for an average day**

17. Meals consumed and source:

|  | Prepared at home | Bought from Office canteen | Bought from other place |
| --- | --- | --- | --- |
| Breakfast |  |  |  |
| Lunch |  |  |  |
| Dinner |  |  |  |
| Snack 1 |  |  |  |
| Snack 2 |  |  |  |
| Snack 3 |  |  |  |

Snacks – number of snacks per day- …….

18. Eating at office:

| Alone |  | in groups |  |
| --- | --- | --- | --- |

19. How many times in the week you had to skip or delay meals at office ………………………..

**Part 04 – Stage of change**

1. Do you follow healthy dietary guidelines such as minimizing refine carbohydrate and sugar and salt consumption and higher fruit and vegetable consumption?
   - 1. If **yes**, for how long?

More than 6 months **(maintenance stage)**

Less than 6 months **(Action stage)**

- - 1. If **no**, are you seriously thinking of changing into healthy diet in next 6 months?
       1. If yes, are you going to change your dietary habits in next month?
          1. If yes **(preparation)**
          2. If no **(contemplation)**
       2. If no **(pre-contemplation)**
