# Supplementary material for: A Stage of Change Theory–Based, Stage-Matched Intervention for Healthy Dietary Intake Among Office Workers in a Low- to Middle-Income Country: Protocol for a Cluster Randomized Trial
Source: JMIR Res Protoc. 2025 Sep 30;14:e70293. doi: 10.2196/70293 (PMC12521855; doi:10.2196/70293)
Supplement: Multimedia Appendix 3 [file resprot_v14i1e70293_app3.pdf]

ASSESSING FOOD INTAKE A VISUAL GUIDE

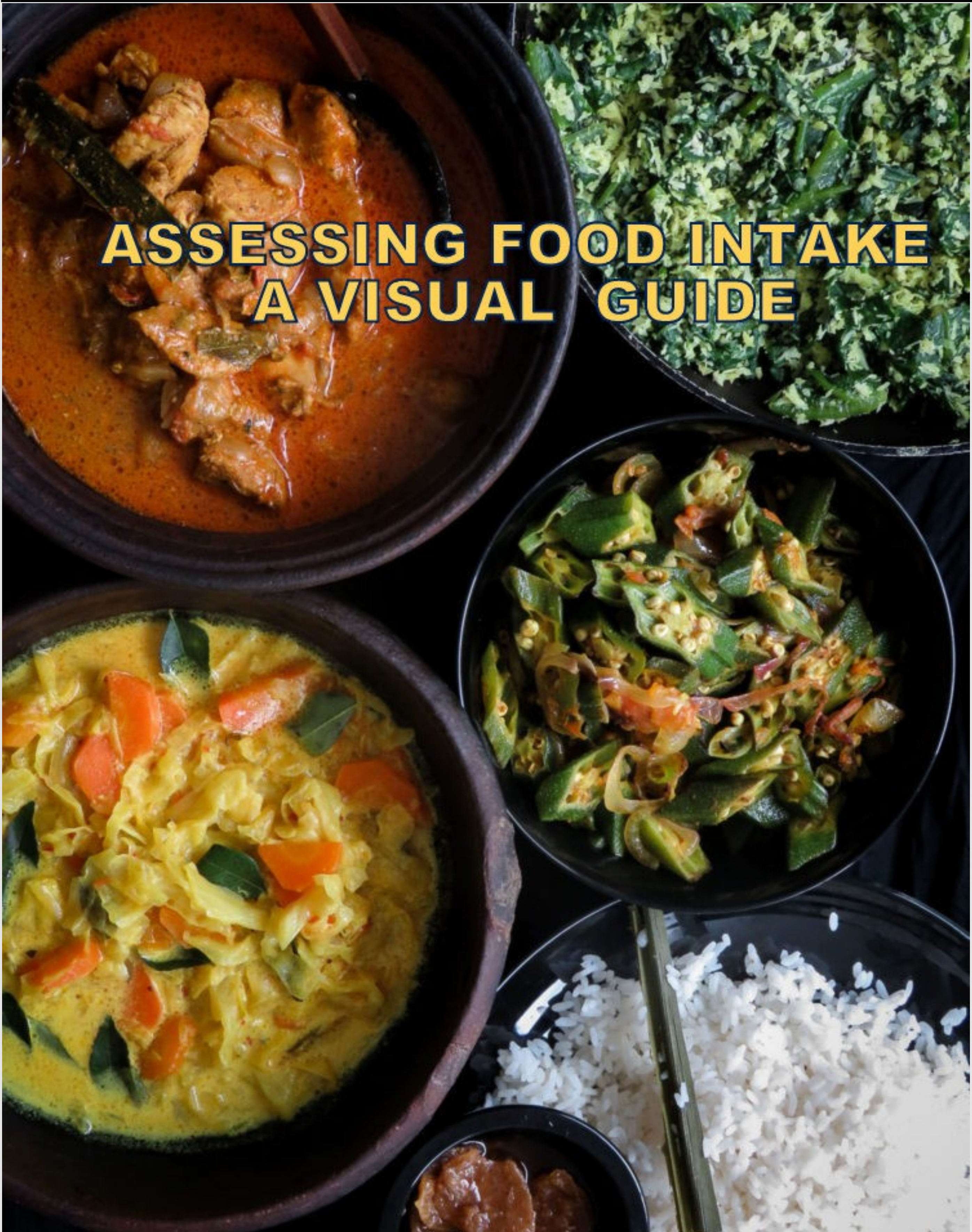

ABBRIVIATIONS

RSC	Curved Rice spoon
RSF	Flat RiceSpoon
TbSL	Large Table Spoon
TbSS	Small Table spoon
TS	Tea spoon
CSL	Large Coconut Shell Spoon
CSM	Medium Coconut Shell Spoon
CSS	Small Coconut Shell Spoon
TC	Tea cup
CCL	Large Commercial Cup
CCS	Small Commercial Cup
DC	Desert Cup

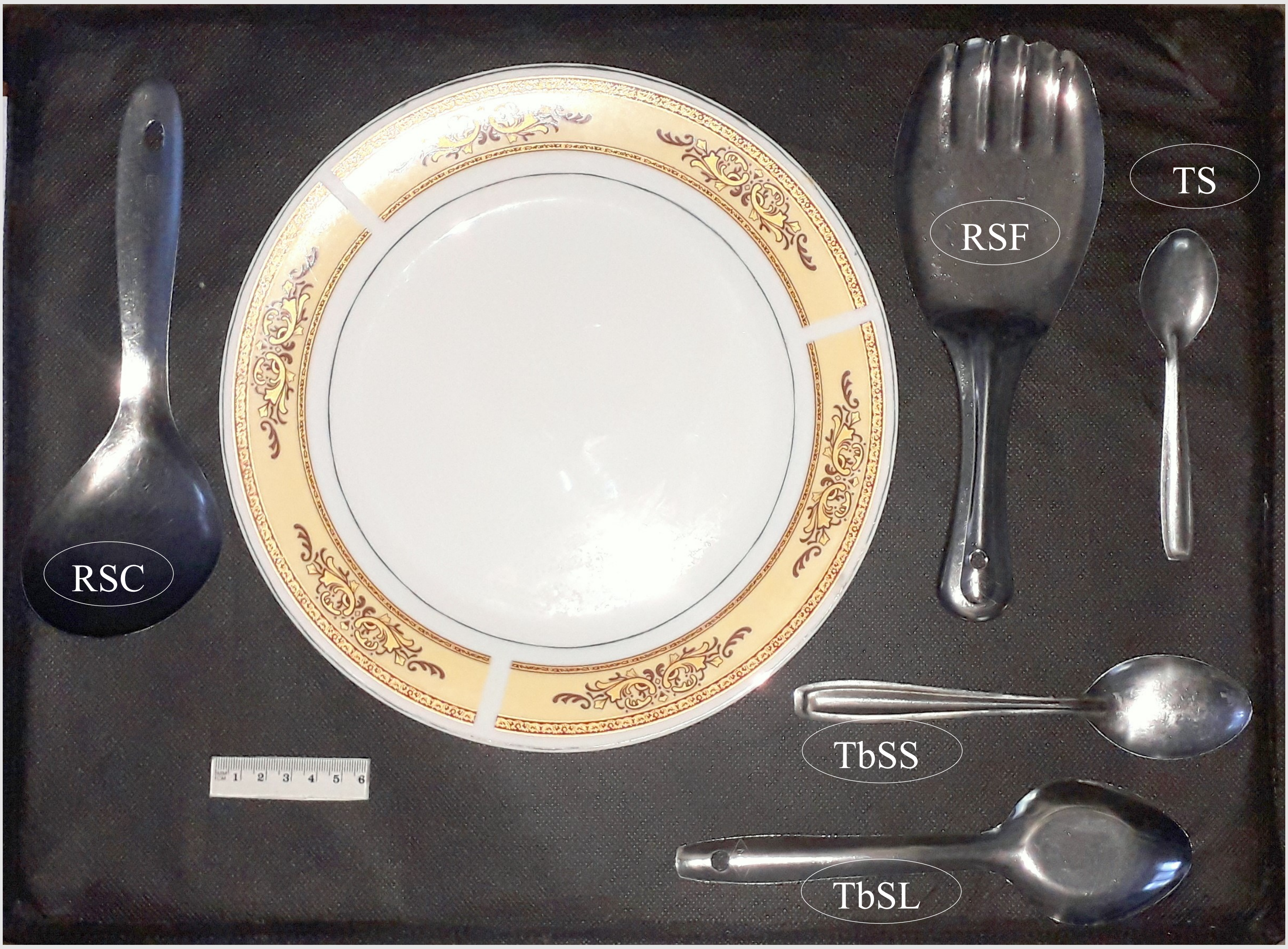

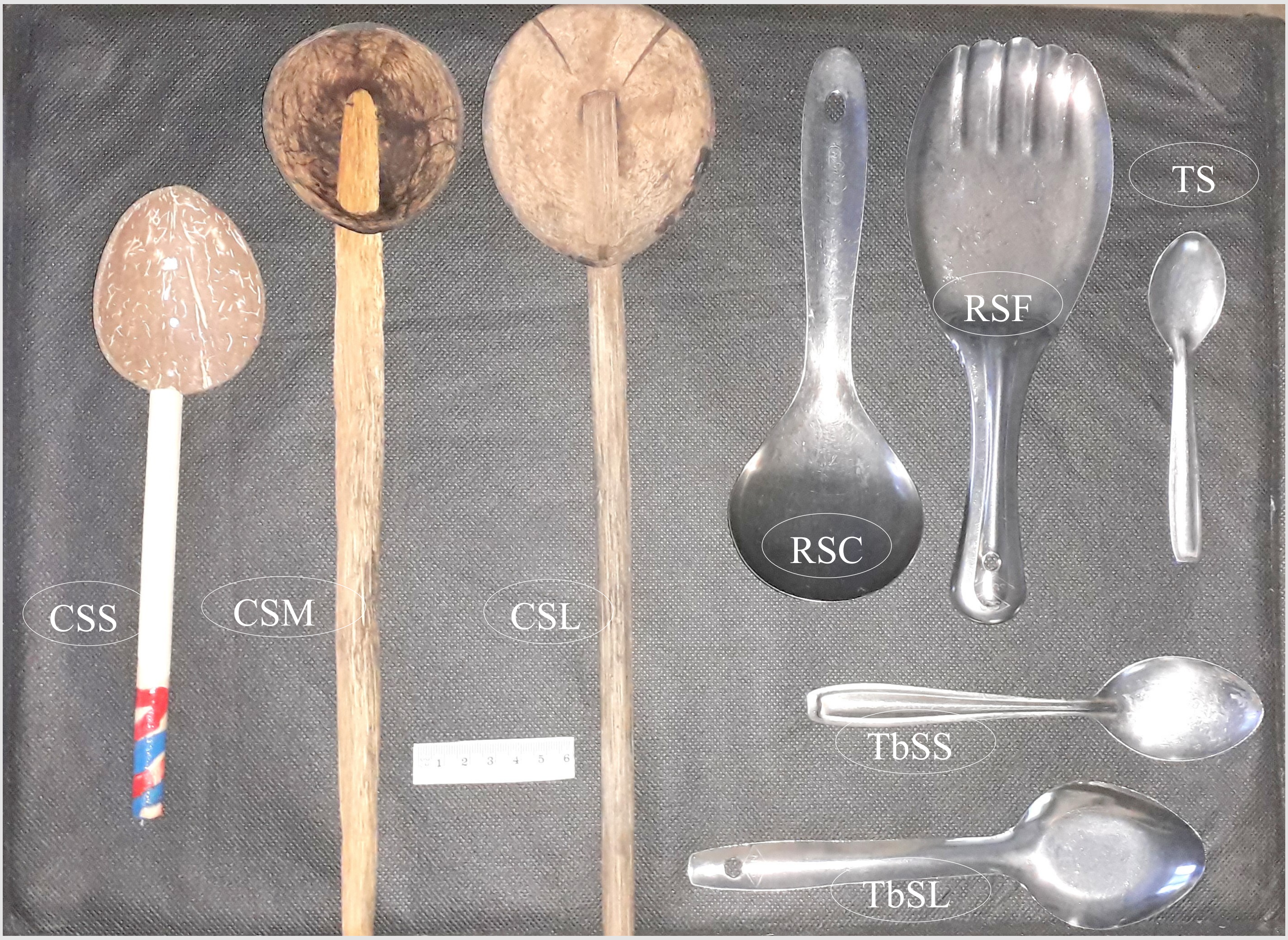

DIFFERENT SIZES OF SPOONS

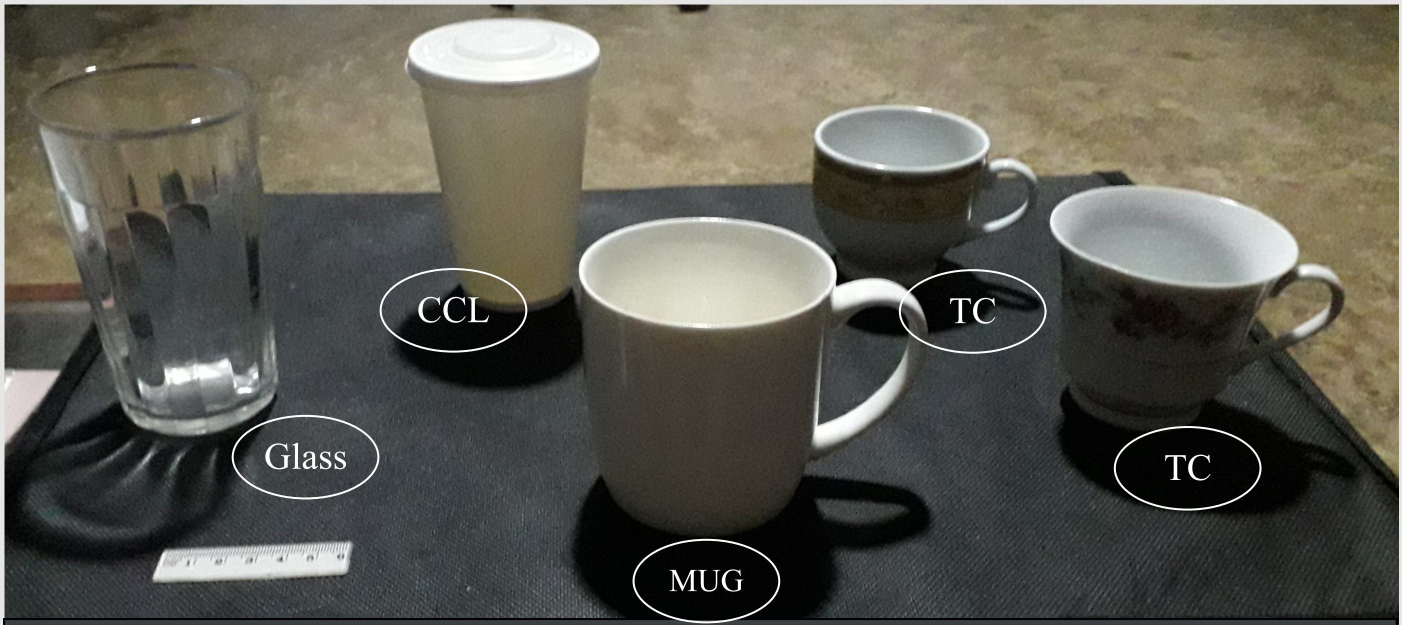

SPOON SIZES CONVERSION (from previous page)

- **RRC =RSF = 4 TbSS**
- **CSL = 4 TbSS**
- **CSM = 3 TbSS**
- **CSS = TbSL = 2 TbSS**

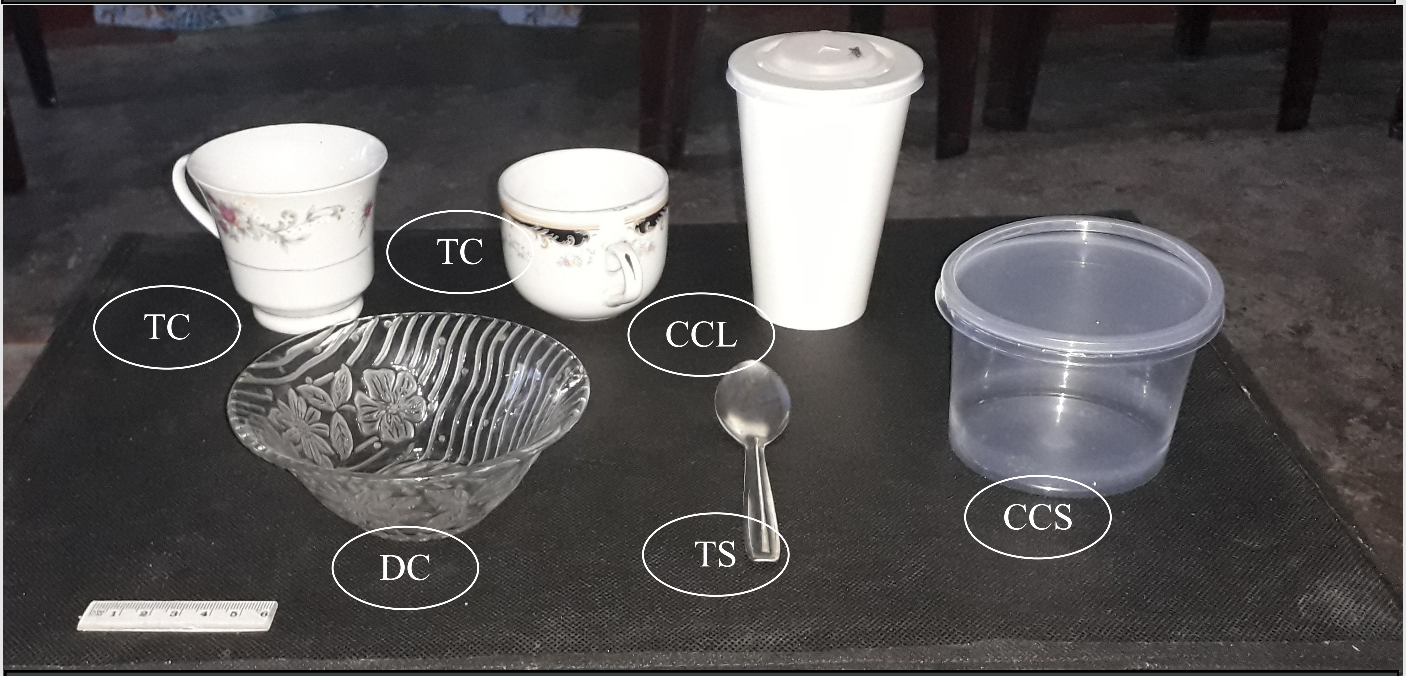

CUP SIZES CONVERSION

- **Glass = CCL = MUG = 400ml**
- **TC = 200ml**
- **DC = TC = 6 TbSS**
- **CCL = 10 TbSS**
- **CCS = 6 TbSS**

DIFFERENT SIZES OF CUPS

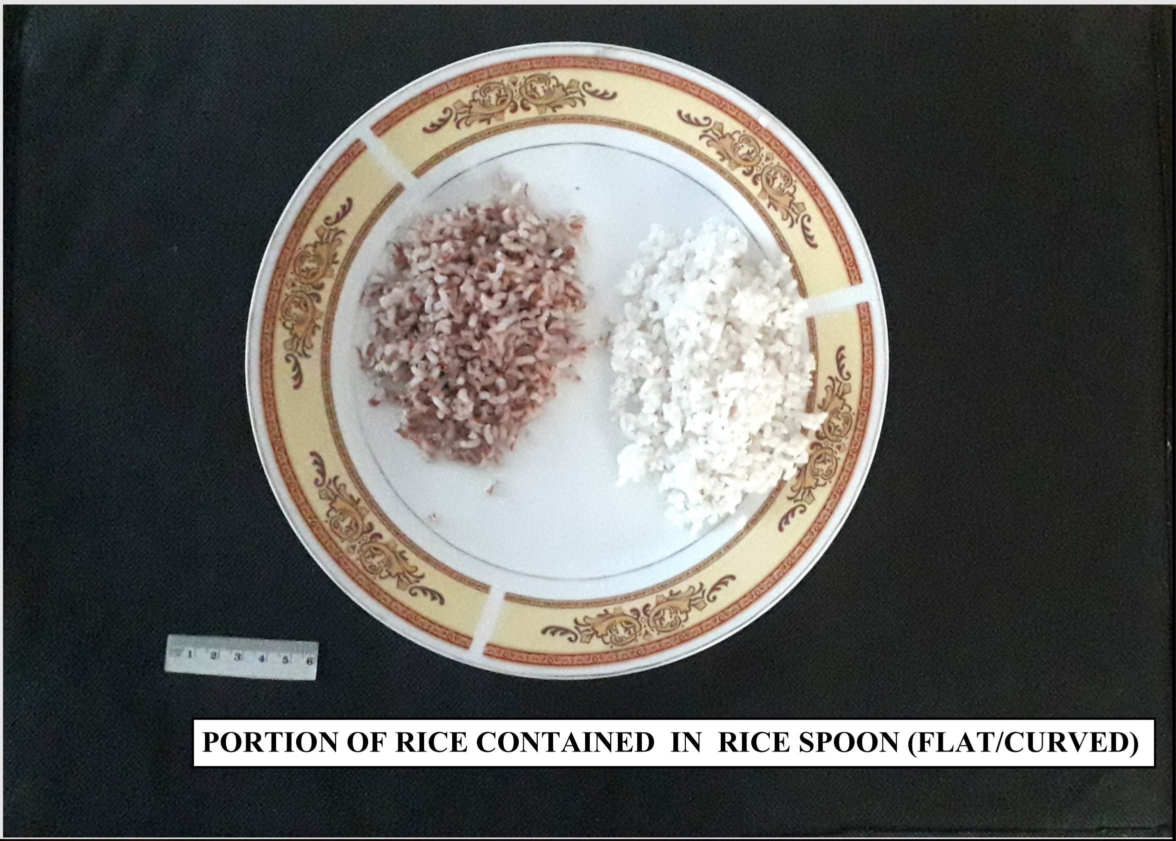

PORTION OF RICE CONTAINED IN RICE SPOON (FLAT/CURVED)

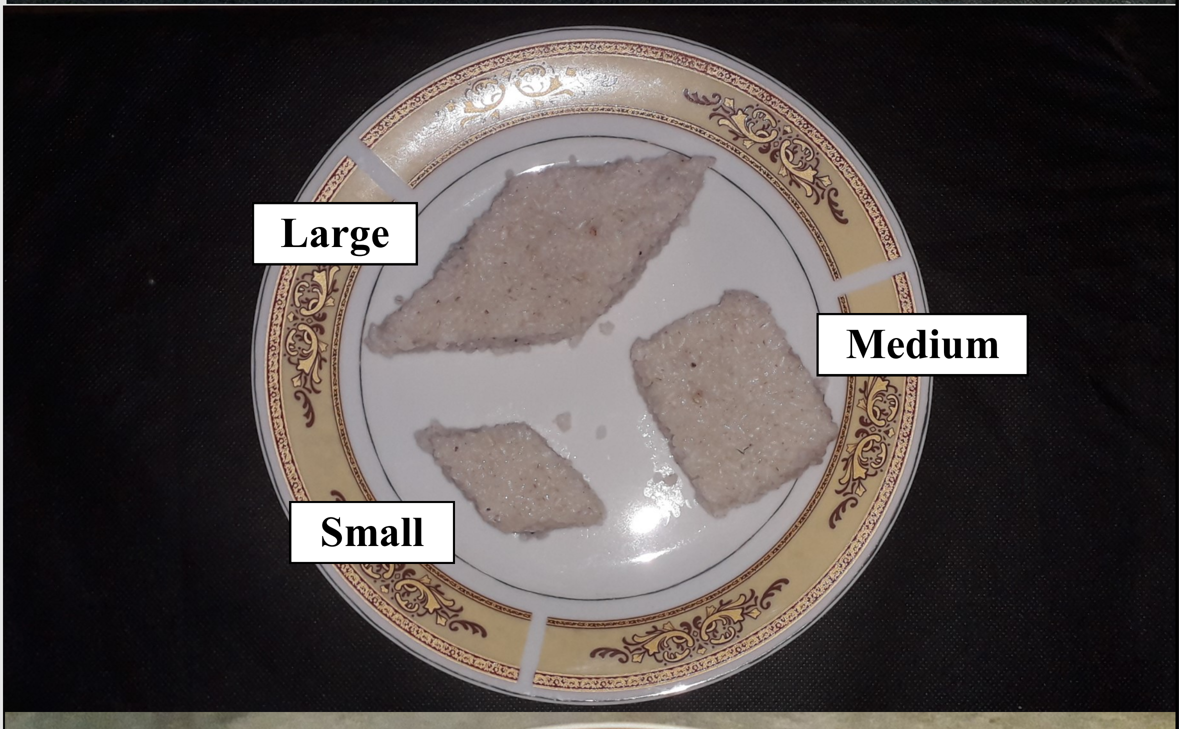

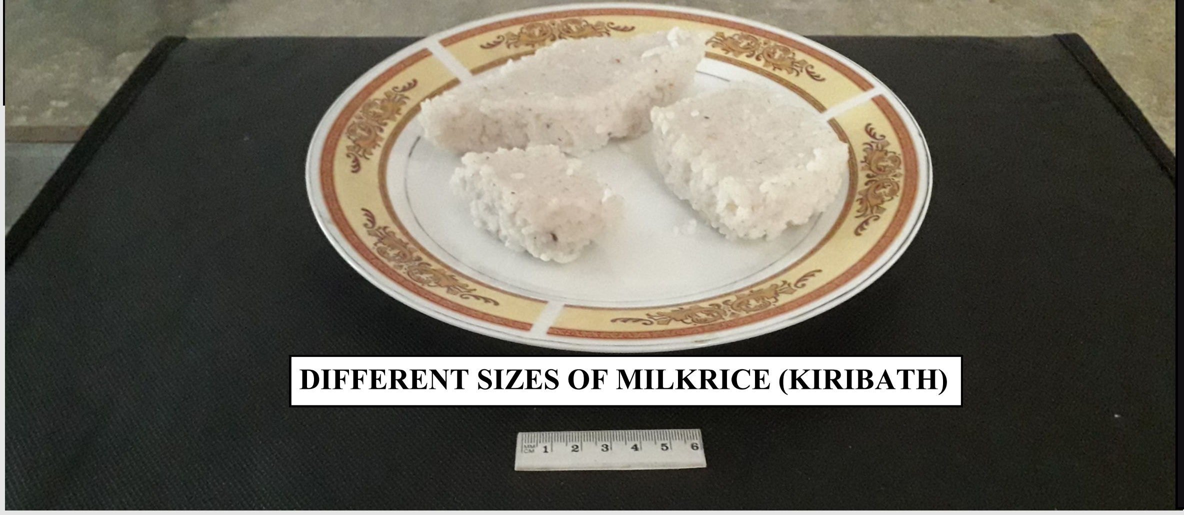

DIFFERENT SIZES OF MILKRICE (KIRIBATH)

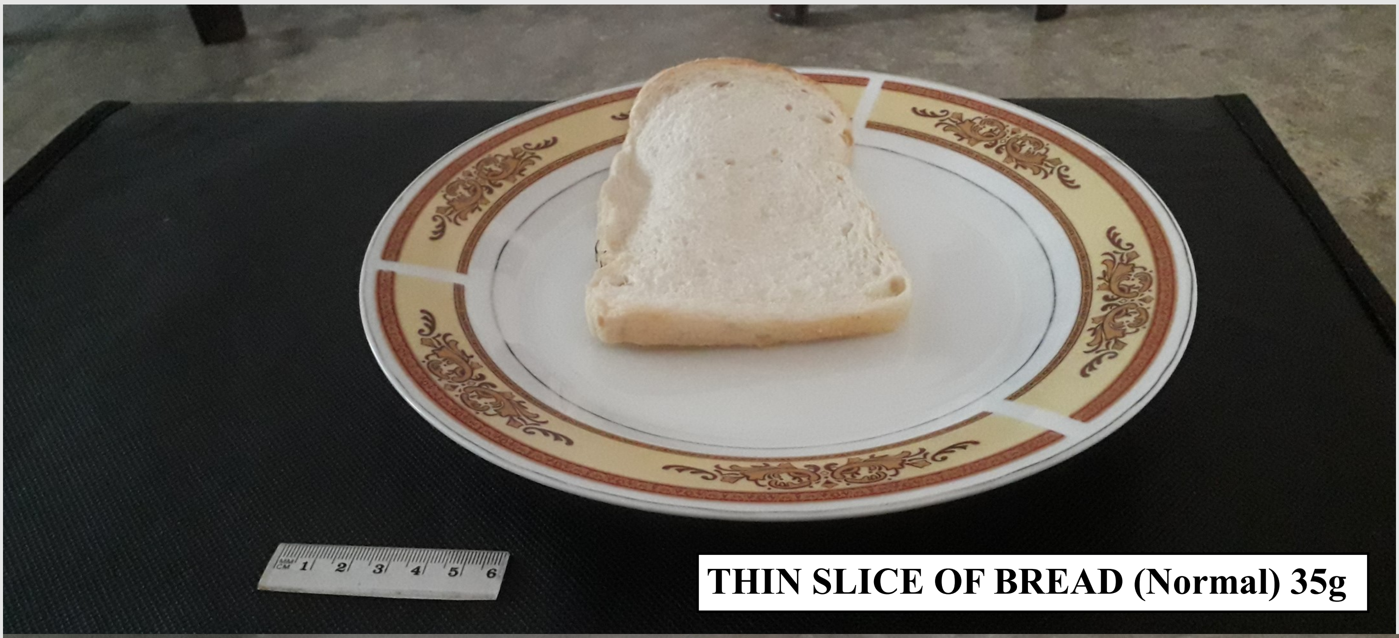
THIN SLICE OF BREAD (Normal) 35g

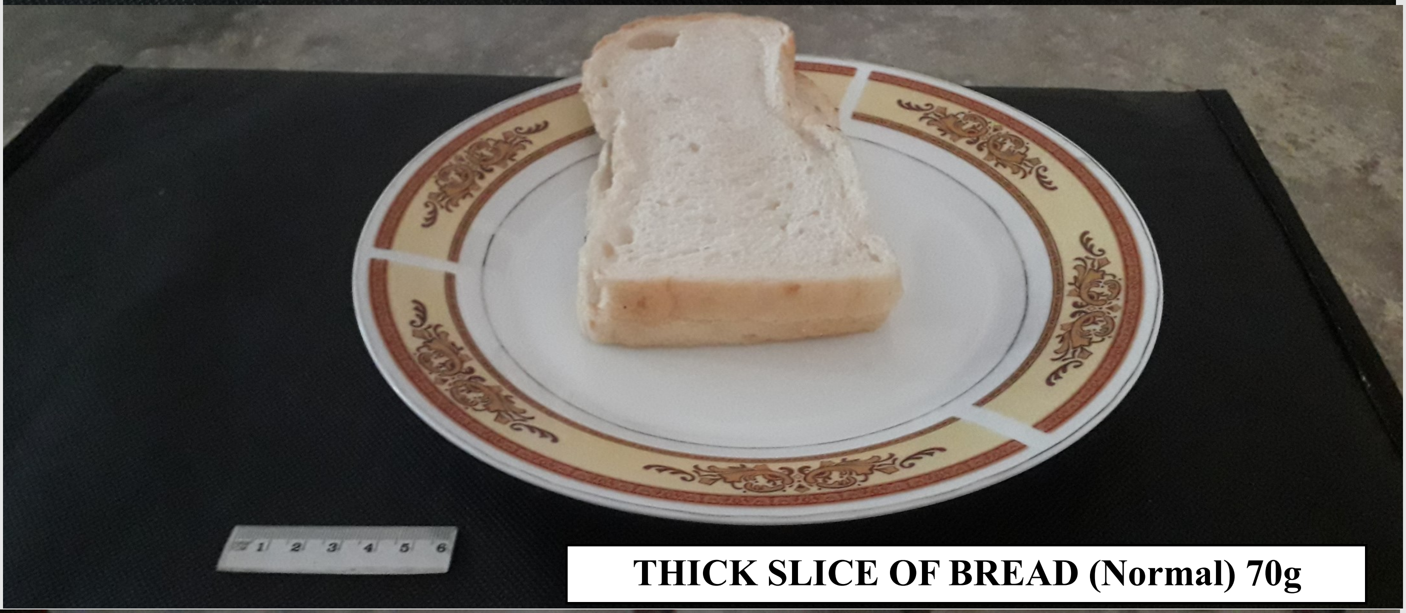
THICK SLICE OF BREAD (Normal) 70g

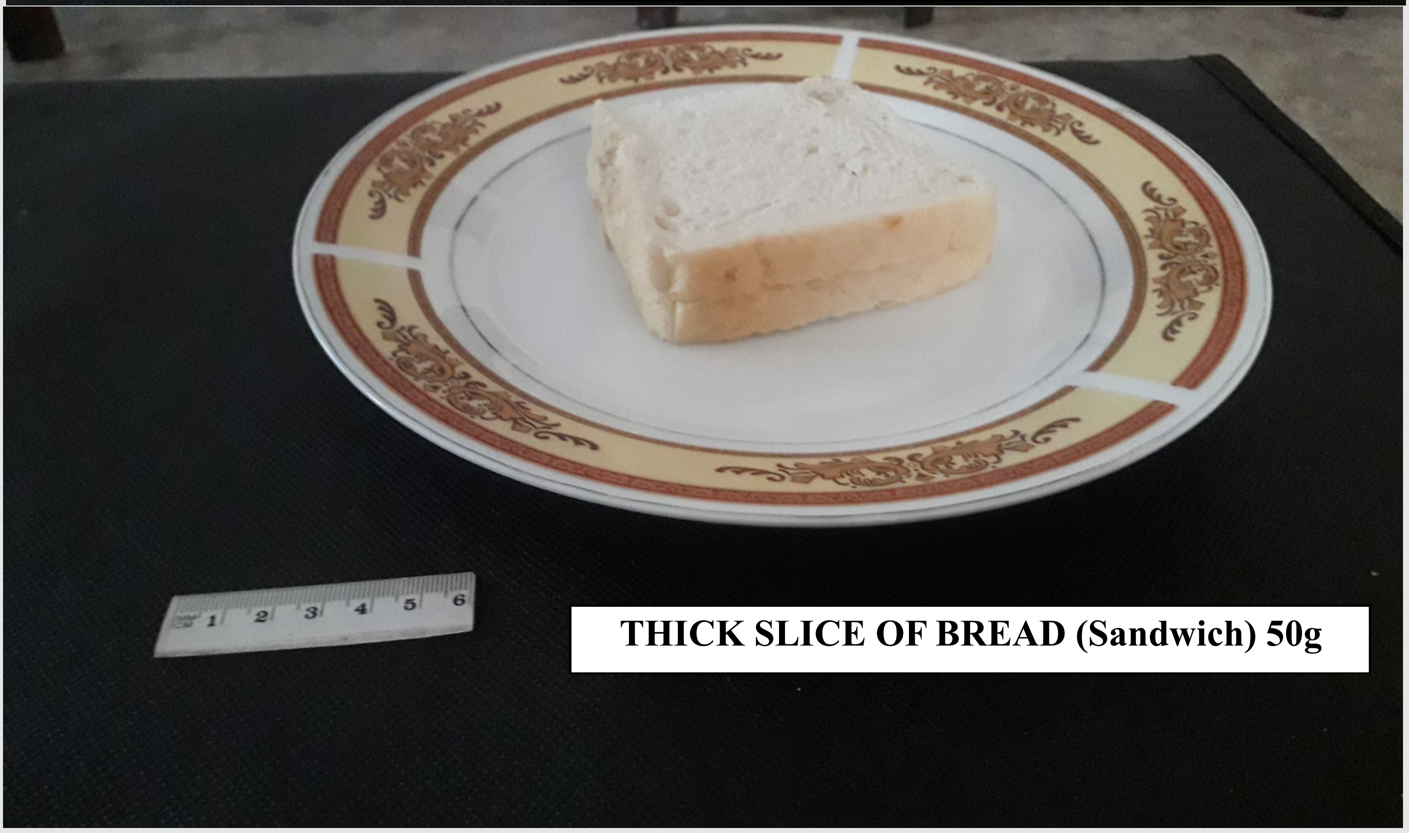
THICK SLICE OF BREAD (Sandwich) 50g

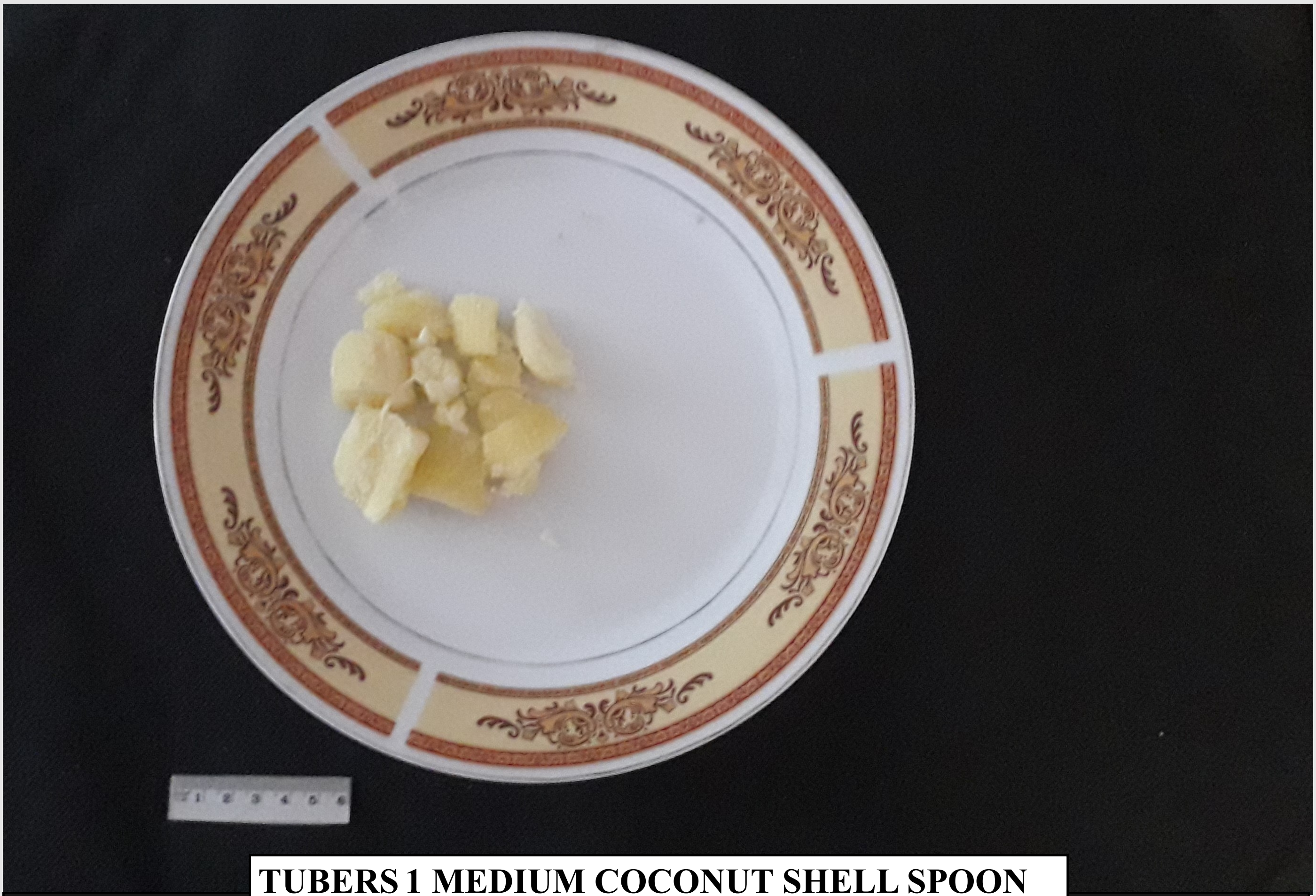

TUBERS 1 MEDIUM COCONUT SHELL SPOON

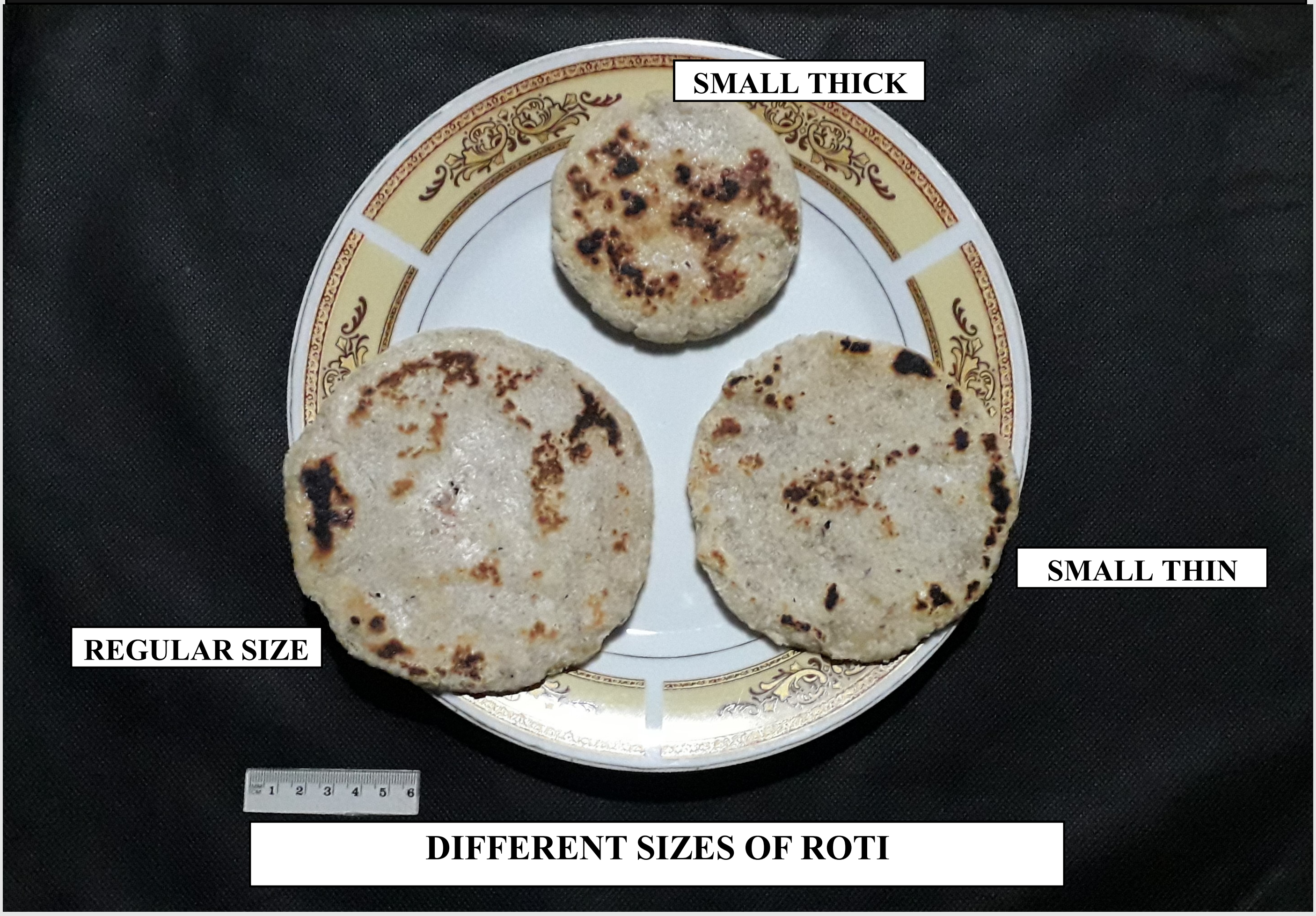

DIFFERENT SIZES OF ROTI

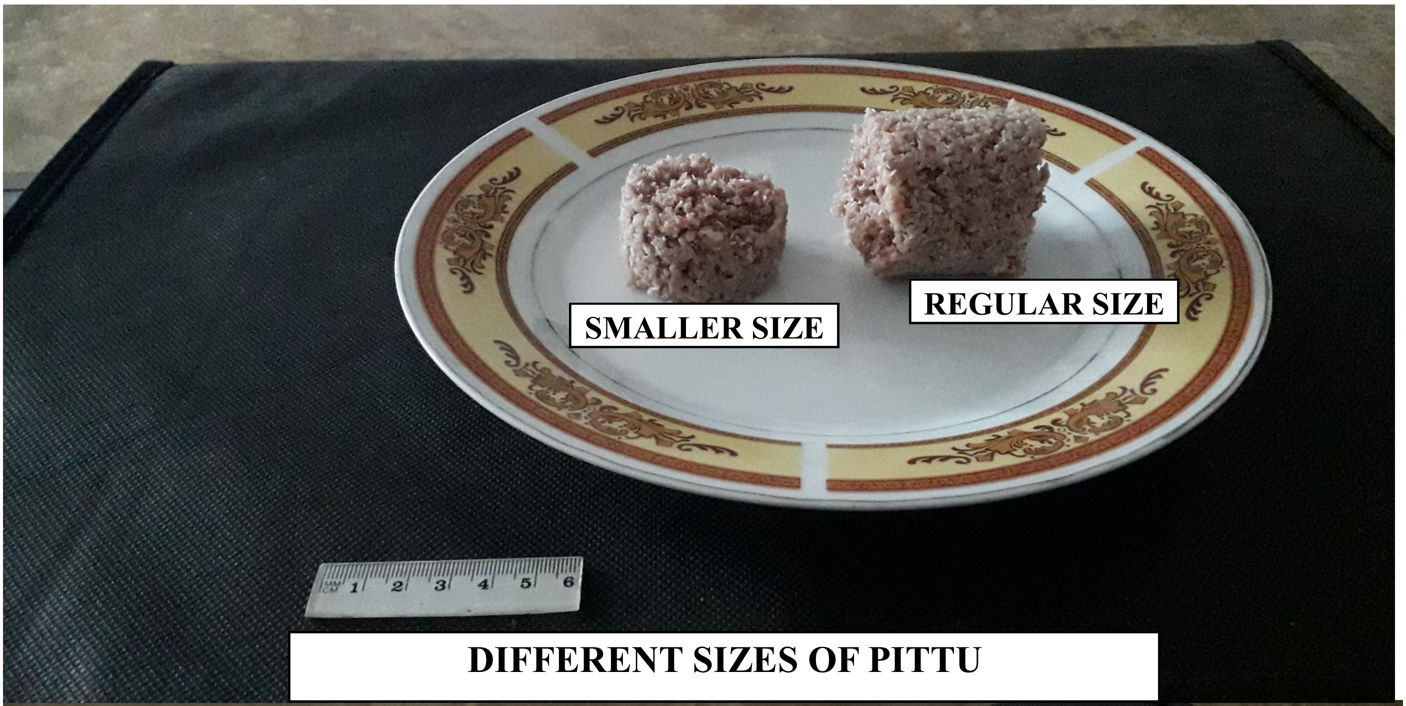
SMALLER SIZE
REGULAR SIZE
DIFFERENT SIZES OF PITTU

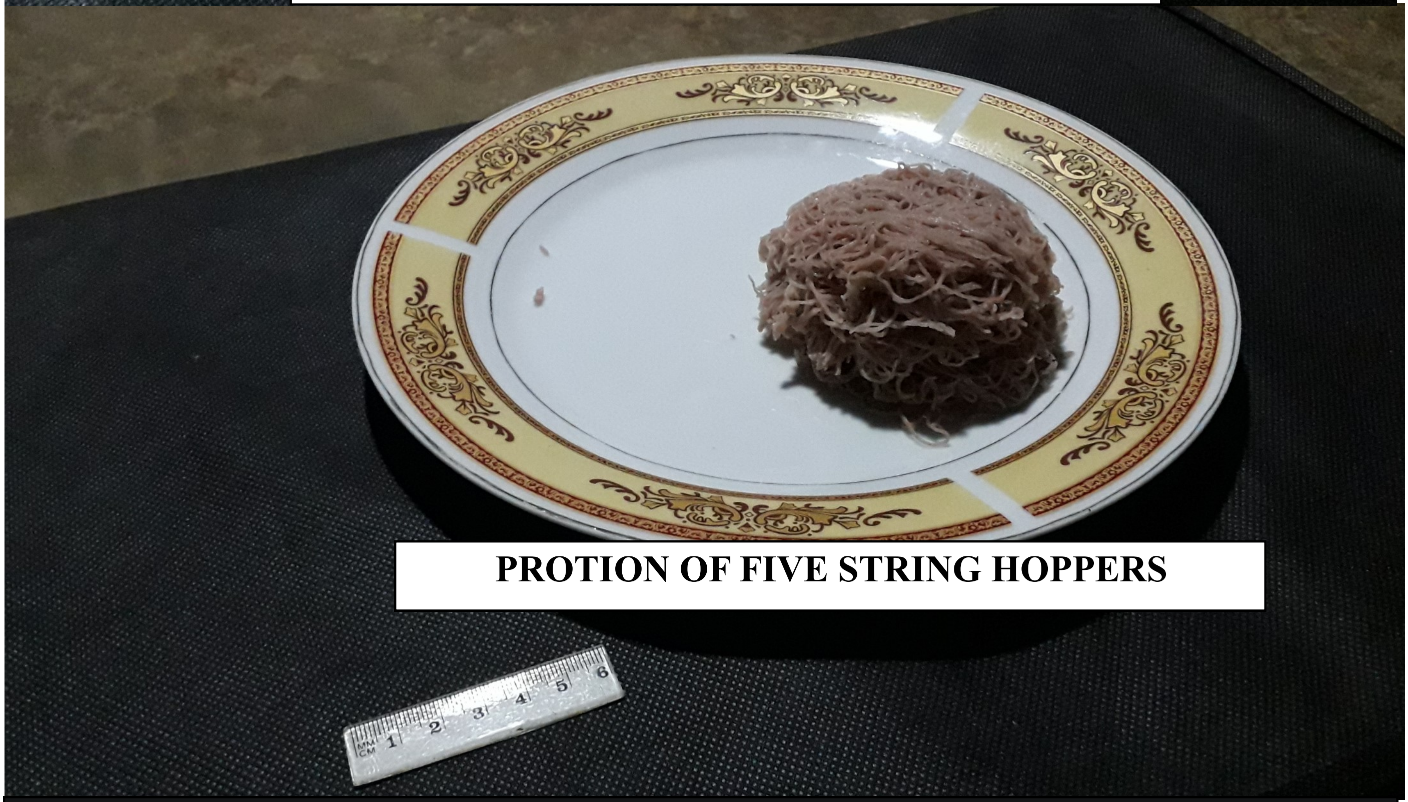
PROTION OF FIVE STRING HOPPERS

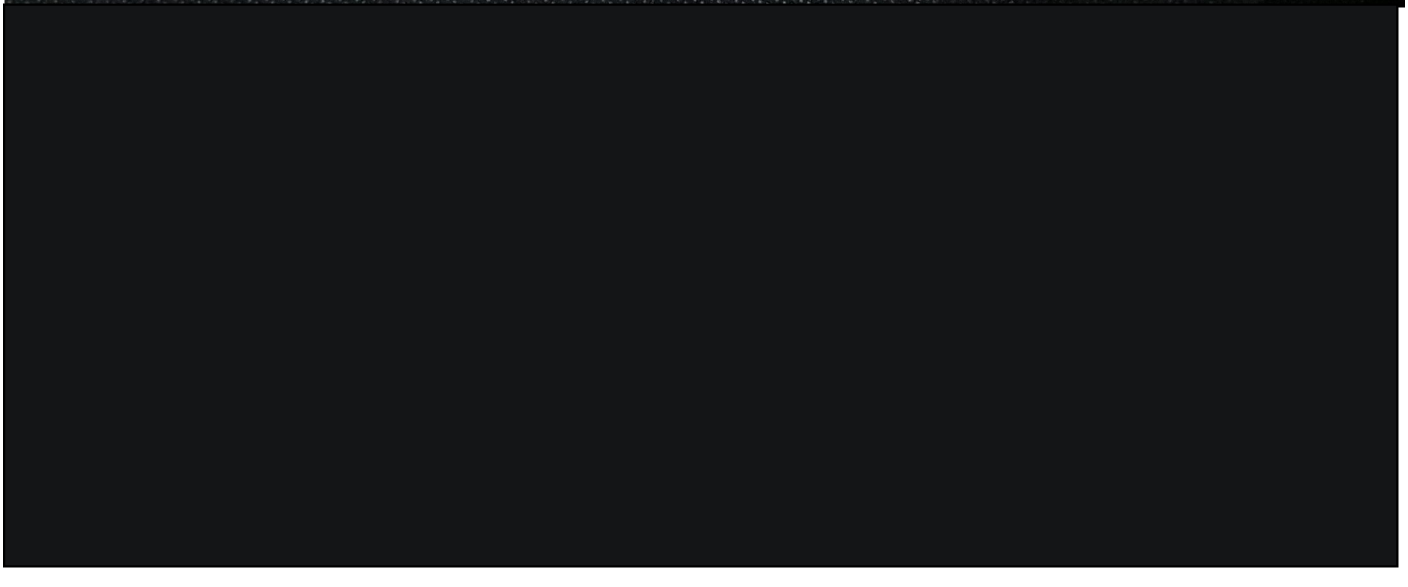

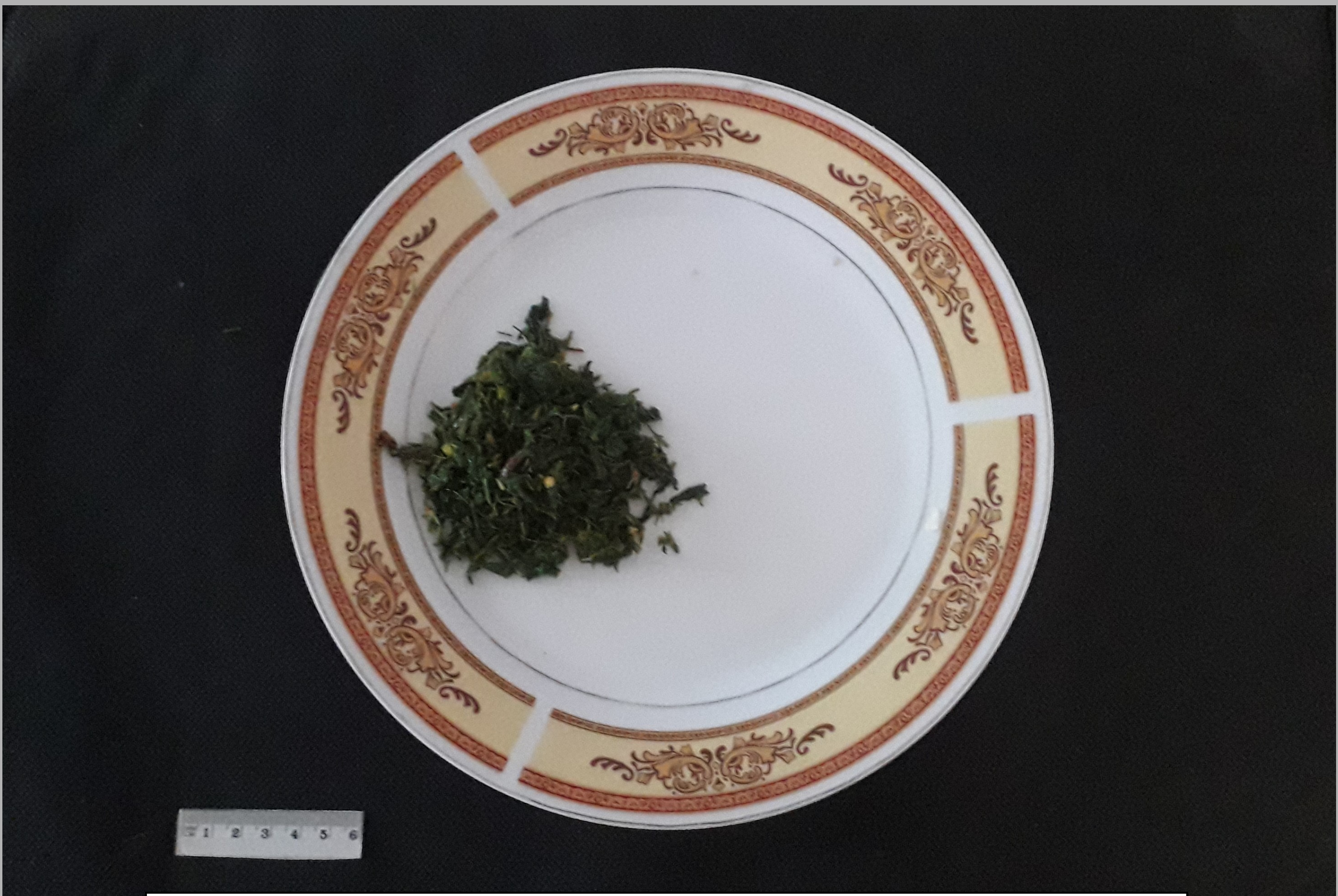

LEAFE VEGETABLE 1 MEDIUM COCONUT SHELL SPOON

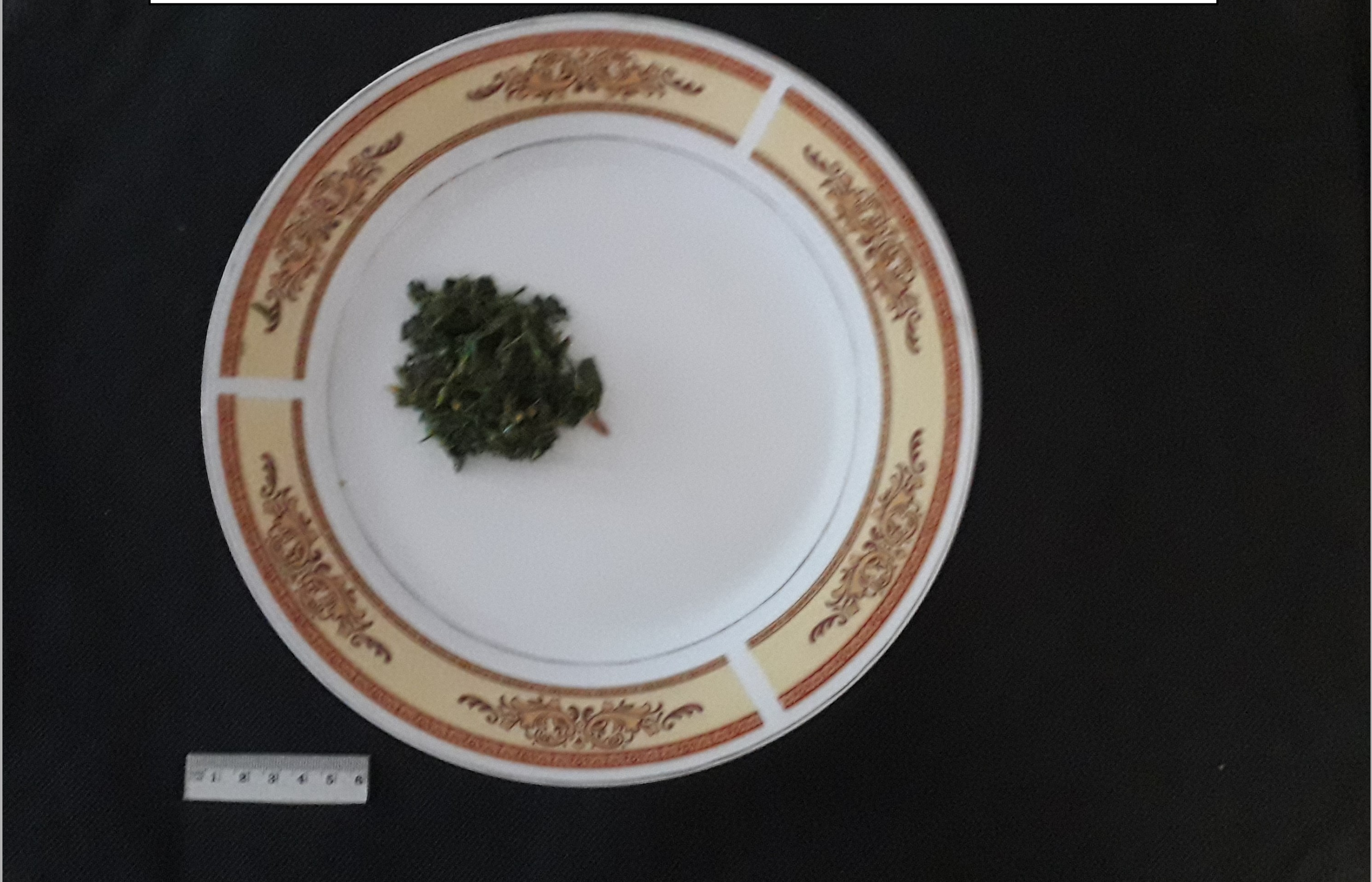

LEAFE VEGETABLE 1 TABLE SPOON

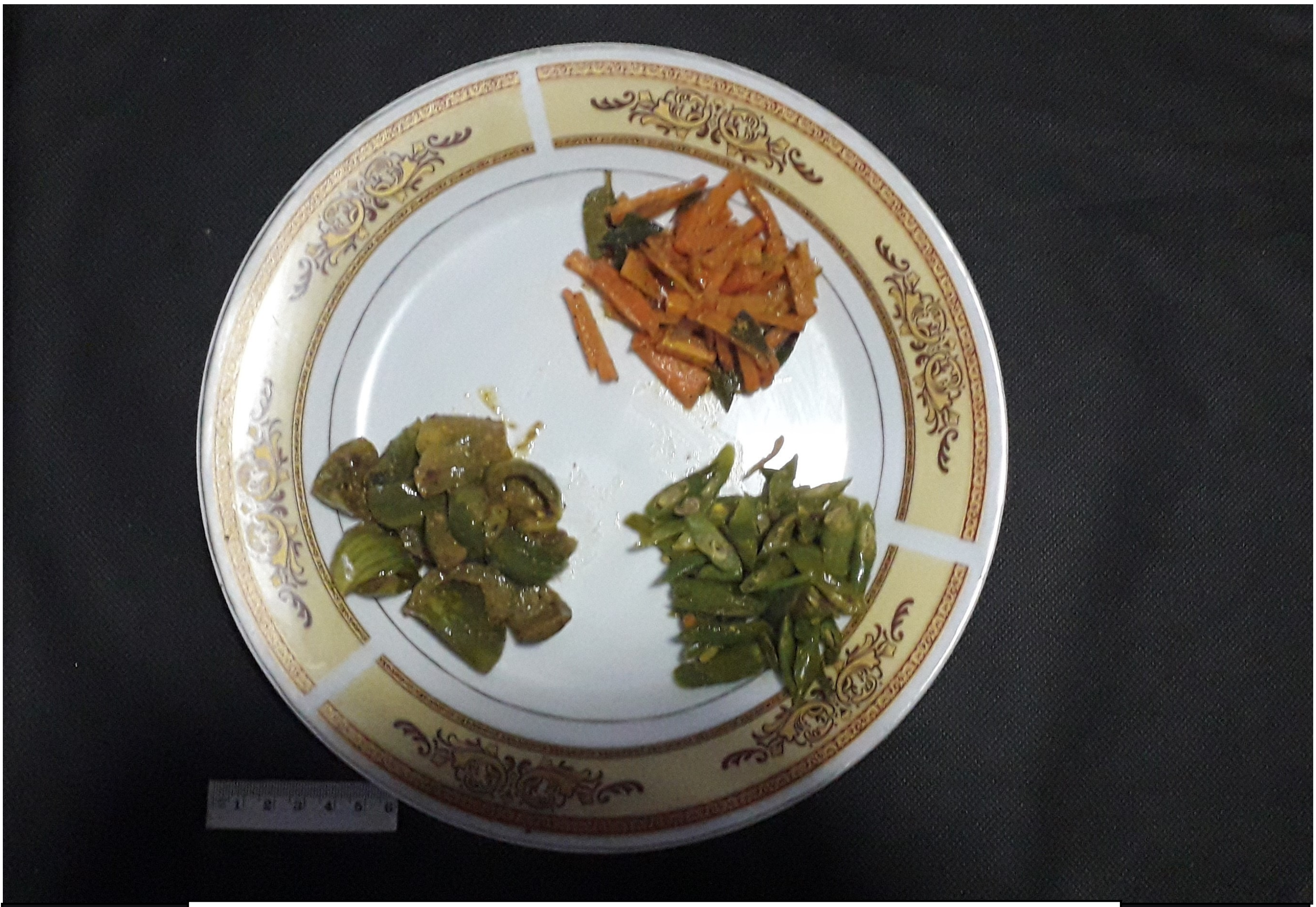

VEGETABLES 1 MEDIUM COCONUT SHELL SPOON

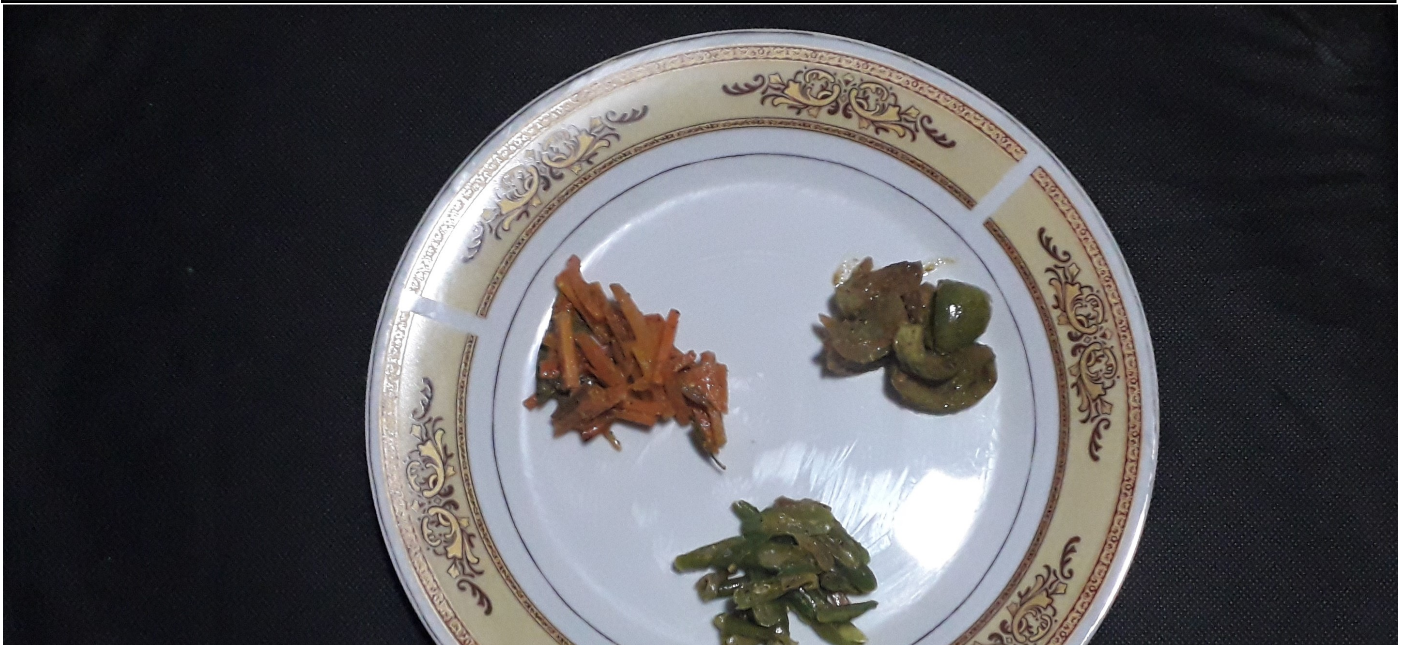

VEGETABLES 1 TABLE SPOON

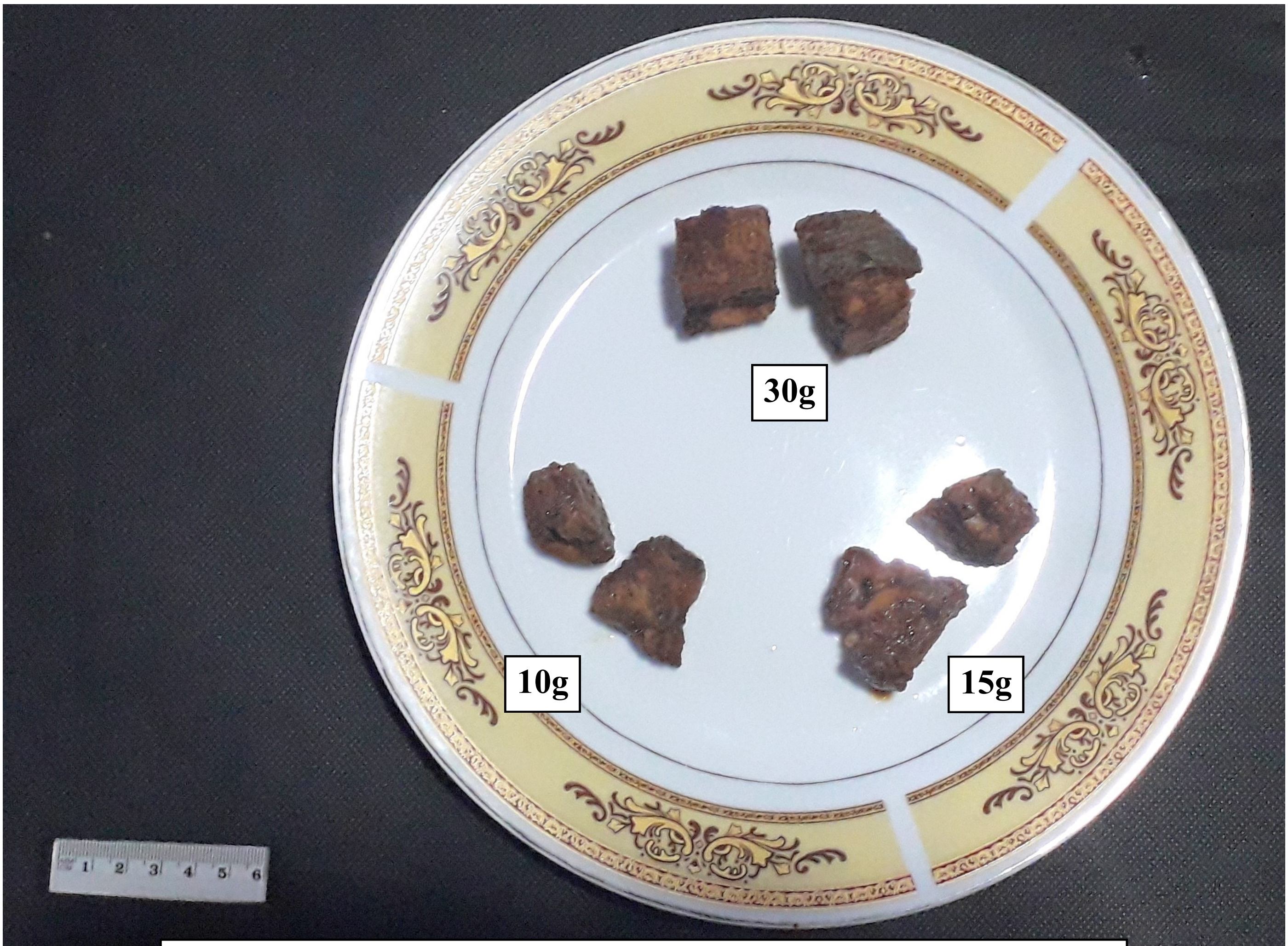

FISH AND MEAT PIECES WITH WEIGHTS

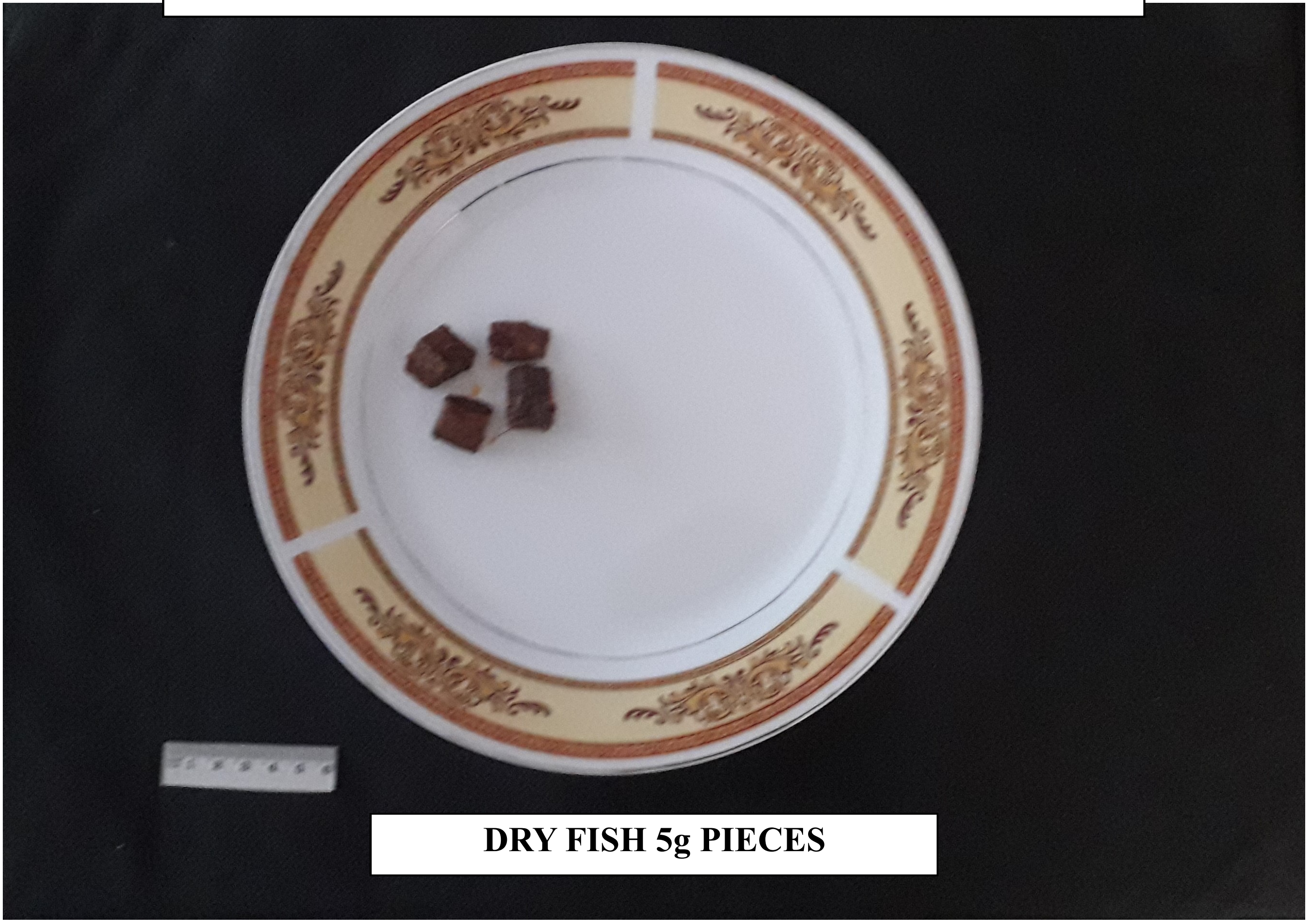

DRY FISH 5g PIECES

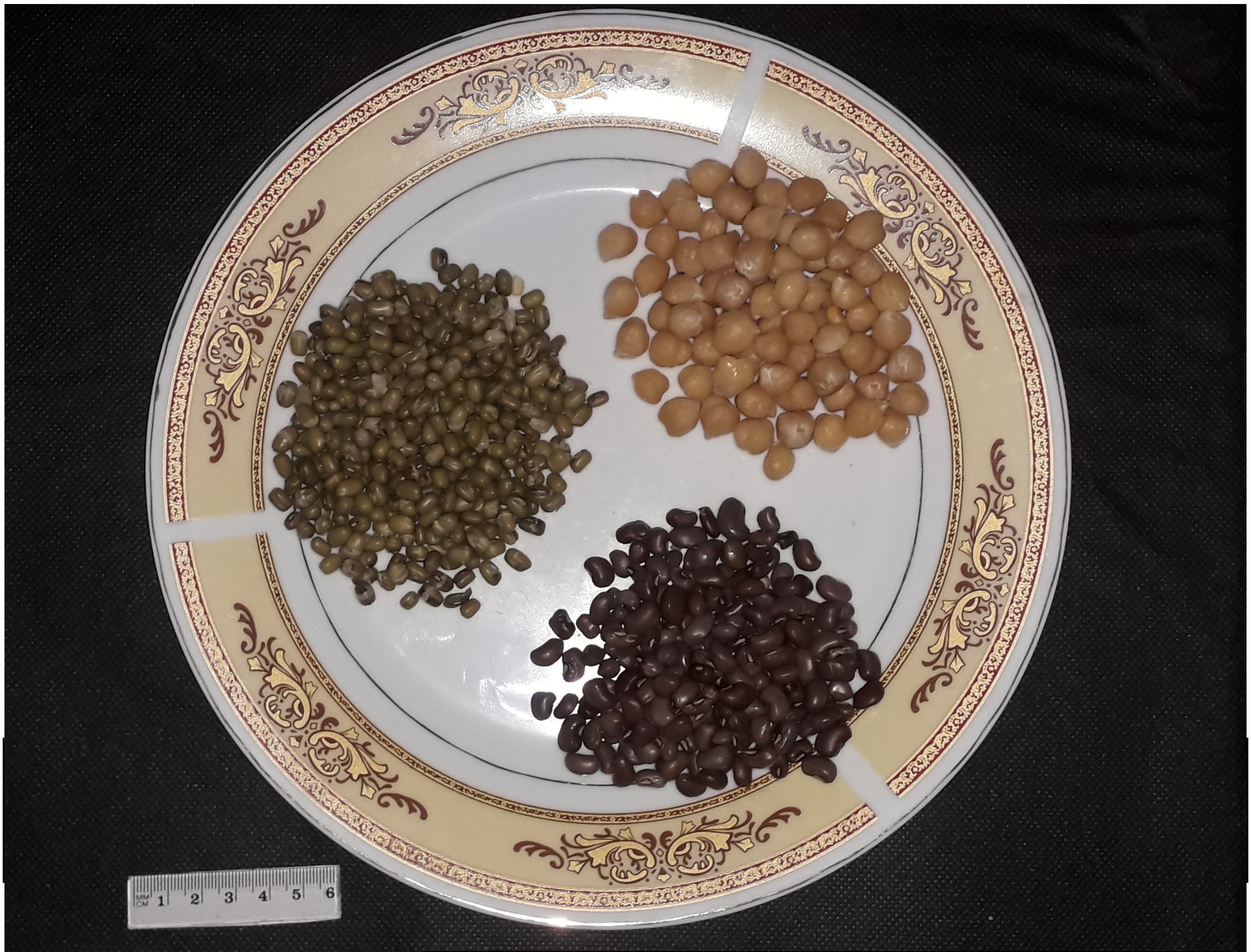

PULSES 1 MEDIUM COCONUT SHELL SPOON

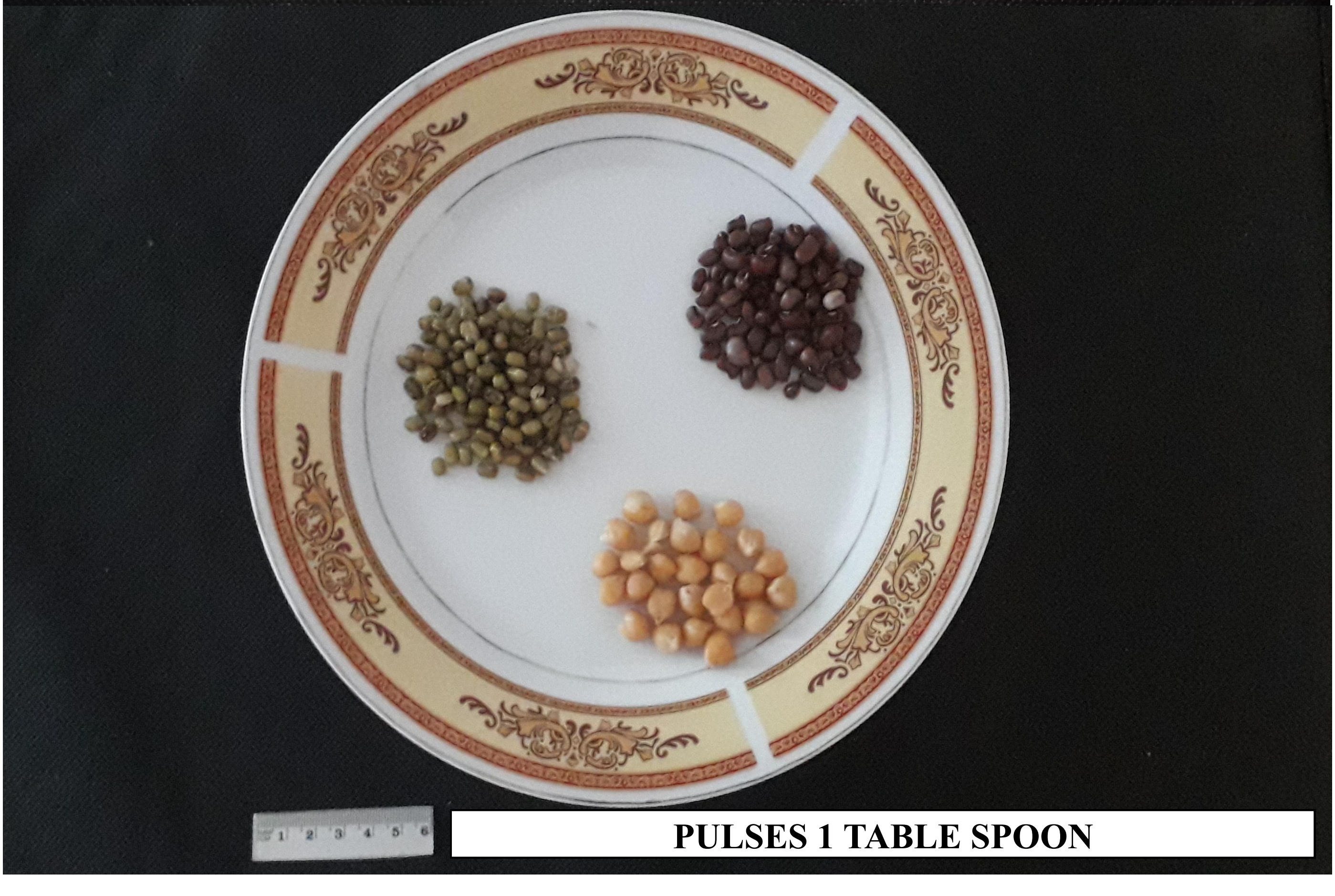

PULSES 1 TABLE SPOON
