# Supplementary material for: A Stage of Change Theory–Based, Stage-Matched Intervention for Healthy Dietary Intake Among Office Workers in a Low- to Middle-Income Country: Protocol for a Cluster Randomized Trial
Source: JMIR Res Protoc. 2025 Sep 30;14:e70293. doi: 10.2196/70293 (PMC12521855; doi:10.2196/70293)
Supplement: Multimedia Appendix 5 [file resprot_v14i1e70293_app5.pdf]

# සෞඛ්‍යමත් ආහාර රටාවක් සඳහා මැදිහත්වීම - 01

# ඔබ ඉන්න තැන.......

- සෞඛ්‍යමත් ලෙස ආහාර ගන්නවා
  - මාස 06 කට වැඩි
  - මාස 06 කට අඩු
- සෞඛ්‍යමත් ලෙස ආහාර රටාව වෙනස් කරගන්න හිතනවා
- සෞඛ්‍යමත් ලෙස ආහාර ගන්න ඕන කියලා දන්නවා
- සෞඛ්‍යමත් ලෙස ආහාර ගන්න ඕන කියලා දන්නෙ නෑ

# ඔබ ඉන්න තැන.......

- සෞඛ්‍යමත් ලෙස ආහාර ගන්නවා
  - මාස 06 කට වැඩි
  - මාස 06 කට අඩු
- සෞඛ්‍යමත් ලෙස ආහාර රටාව වෙනස් කරගන්න හිතනවා

- සෞඛ්‍යමත් ලෙස ආහාර ගන්න ඕන කියලා දන්නවා
- සෞඛ්‍යමත් ලෙස ආහාර ගන්න ඕන කියලා දන්නෙ නෑ

# බෝනොවන රෝග

- හෘදයාබාධ
- ආඝාතය (අංශභාගය) 40%
- පිළිකා 10%
- දියවැඩියාව 7%
- ශ්වසන පද්ධතිය සම්බන්ධ රෝග 8%
- වෙනත් 10%

# වැලකී සිටින්නේ කෙසේද?

- මත් පැන් පානයෙන් වැලකීම
- දුම්පානයෙන් සහ දුම්කොළ භාවිතයෙන් වැලකීම
- **සෞඛ්‍යමත් ආහාර**
- ක්‍රියාශීලී දිවිය

# සෞඛ්‍යමත් ආහාර (Healthy Diet)

- නියමිත උස සහ බර
- බෝ නොවන රෝග වලක්වා ගැනීම
  - අධිරුධිර පීඩනය
  - දියවැඩියාව
  - කොලෙස්ටරෝල්
  - පිළිකා

# Deaths attributable to risk factors by income groups

NCD risk factors account for large number of deaths in all income groups

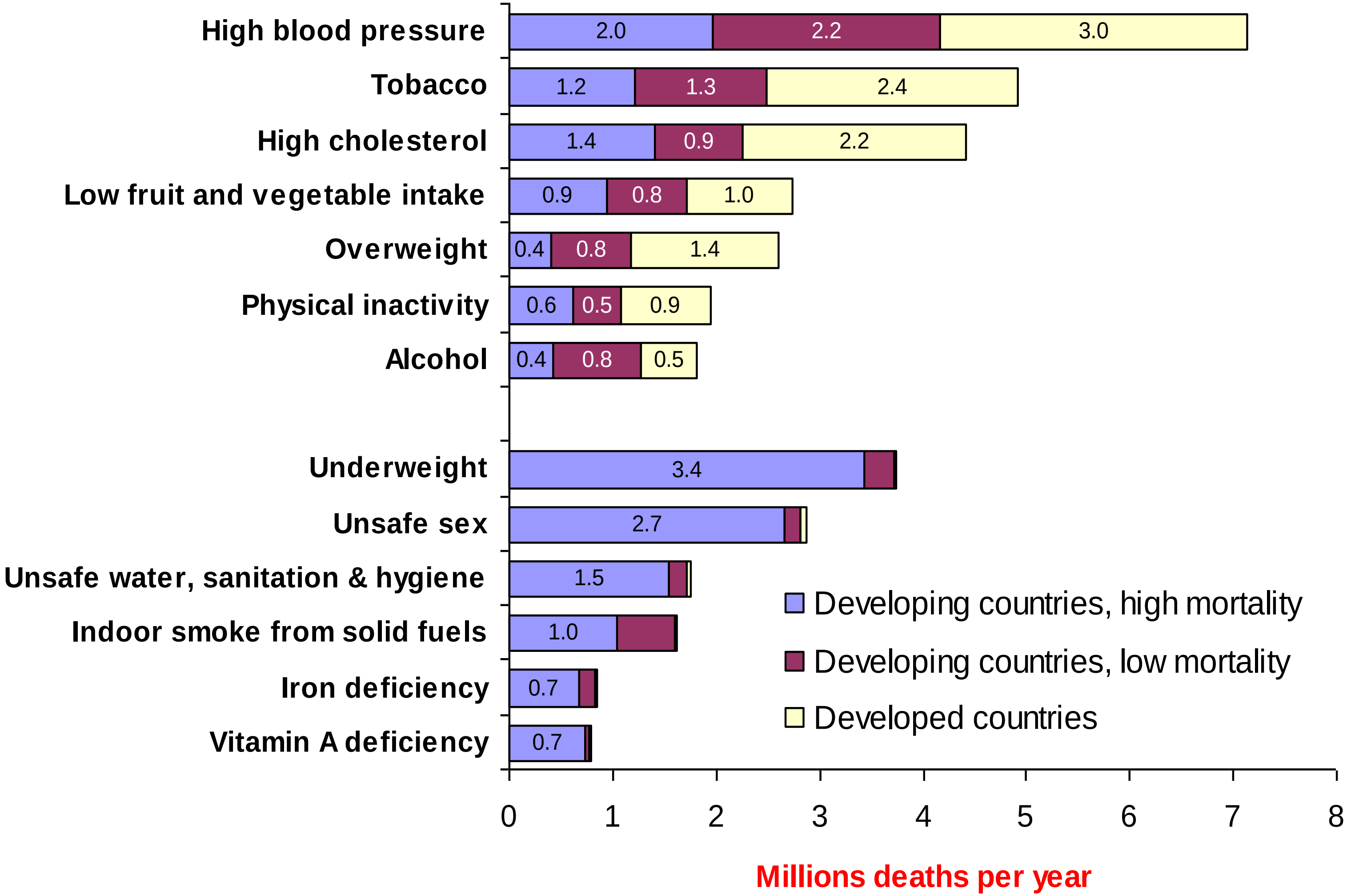


World Health Report, WHO, 2002

# බෝ නොවන රෝග වල බලපෑම

- තනි පුද්ගලයකුට, පවුලට හෝ සමාජයට / රටට
- ආර්ථික
- සාමාජීය
- සංස්කෘතික

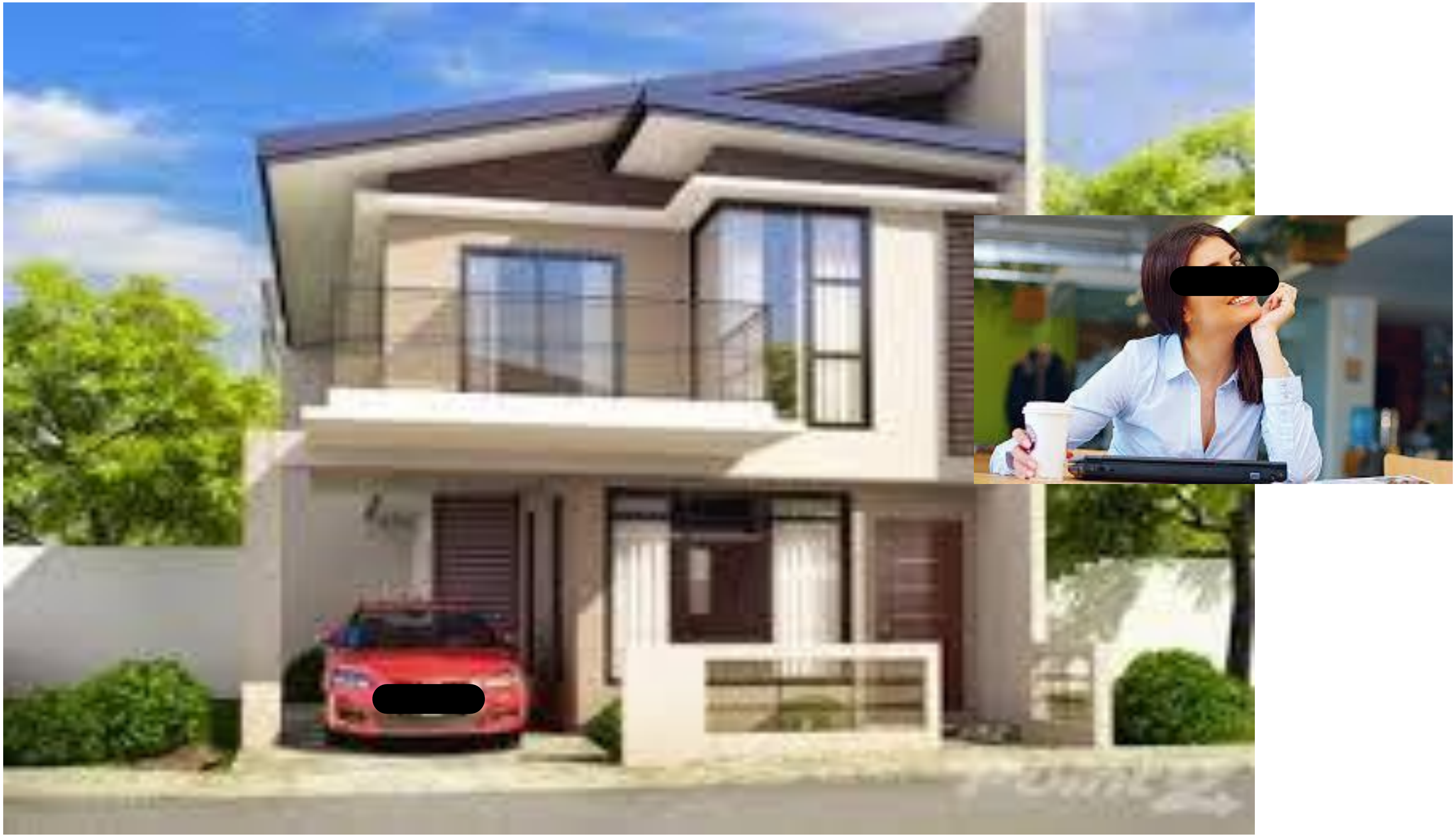

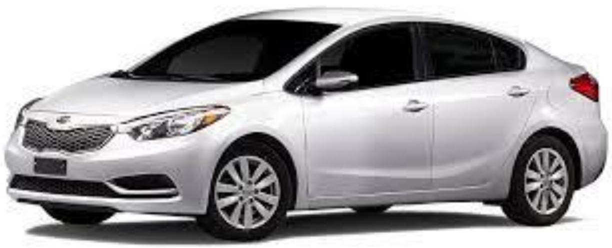

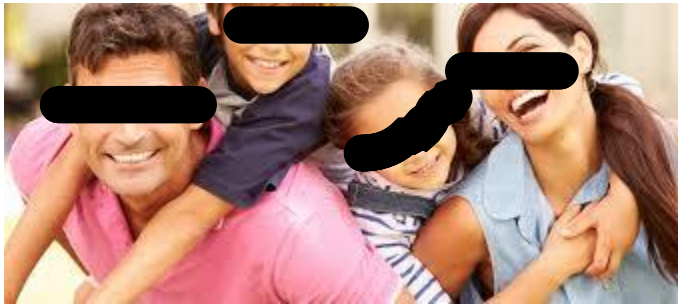

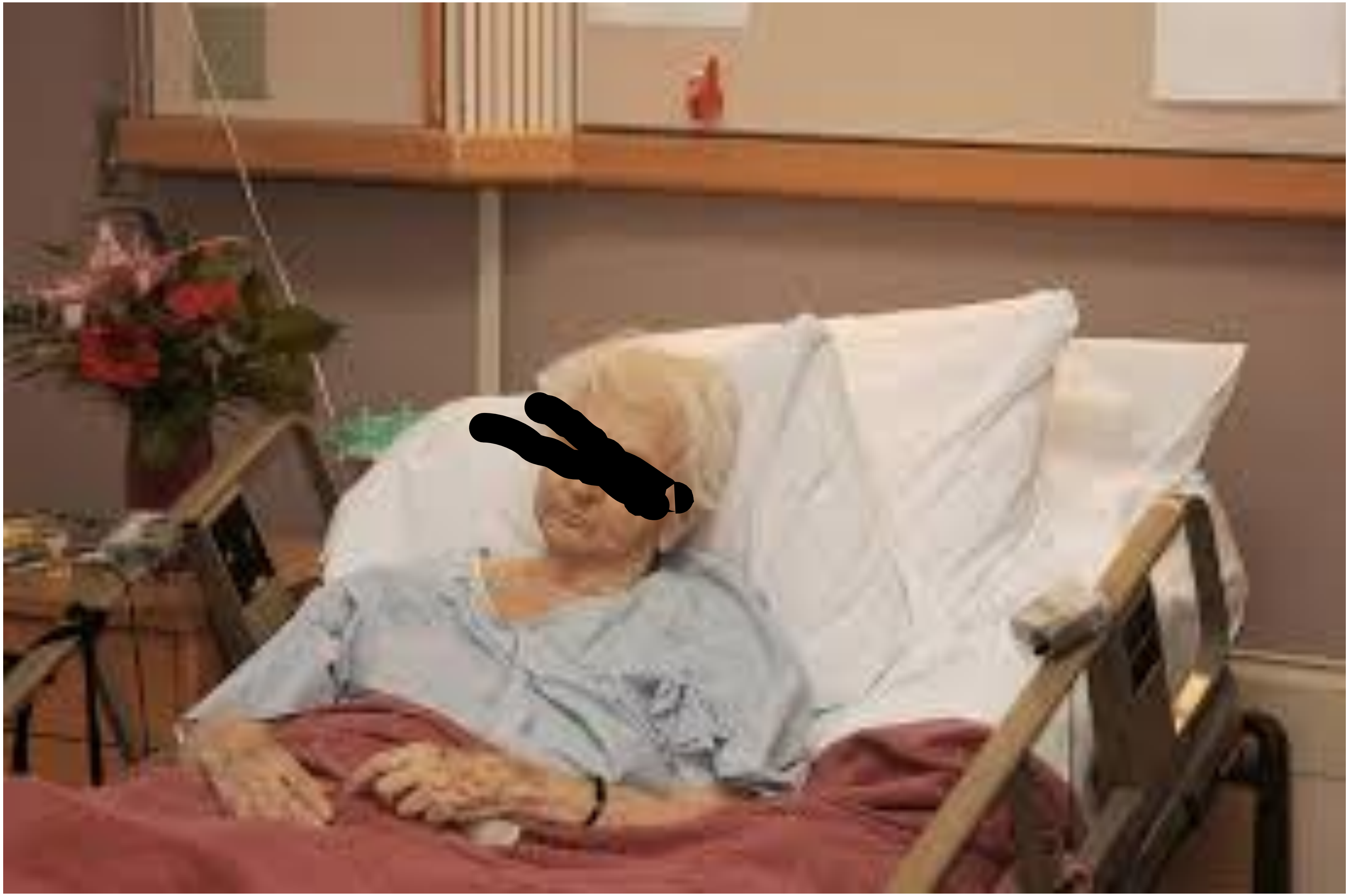

මතු සම්බන්ධයි......
