# Supplementary material for: A Stage of Change Theory–Based, Stage-Matched Intervention for Healthy Dietary Intake Among Office Workers in a Low- to Middle-Income Country: Protocol for a Cluster Randomized Trial
Source: JMIR Res Protoc. 2025 Sep 30;14:e70293. doi: 10.2196/70293 (PMC12521855; doi:10.2196/70293)
Supplement: Multimedia Appendix 6 [file resprot_v14i1e70293_app6.pdf]

සෞඛ්‍යමත් ආහාර රටාවක් සඳහා මැදිහත්වීම - 02

වෙනස් වෙන්න පුළුවන් (ද?)

	වෙනස් වීමට උදව් කරයි	වෙනස් වීමට බාධා කරයි
අප තුල පවතින සාධක	ශක්තීන්	දුර්වලතා
බාහිර සාධක	අවස්ථා	බාධා

ශක්තීන්

- කාබෝහයිඩ්‍රේට (බත්) අඩු ප්‍රධාන ආහාර වේල
- නිවසේ පිළියෙල කල ආහාර
- දැනුම්වත් බව / තොරතුරු සෙවීම
- වෙනස් වීමට කැමැත්ත

දුර්වලතා

- සෞඛ්‍යමත් නොවන අමතර ආහාර බාවිතය
- එළවළු පළතුරු අඩුවෙන් ආහාරයට ගැනීම
- ස්වයං විනය අඩු බව
- ? කාලය ප්‍රමාණවත් නොවීම

අවස්ථා

- දැනුම්වත් වීමට ඇති අවස්ථා
- සෞඛ්‍යමත් ආහාර වෙත යොමුවීමේ ප්‍රවණතා
- ආහාර සම්බන්ධ නීතිමය තත්ව
  - සීනි ප්‍රමාණය දැක්වීම
- කණ්ඩායම් වශයෙන් ආහාර ගැනීම
- එළවළු පළතුරු සුලබ වීම

බාධා

- තොරතුරු වල නිරවද්‍යතාවය පිළිබඳ ගැටළු
- සෞඛ්‍යමත් නොවන ආහාර සහිත ආපන ශාලාව
- සෞඛ්‍යමත් අමතර ආහාර නොමැති වීම

මතු සම්බන්ධයි......
