# Supplementary material for: A Stage of Change Theory–Based, Stage-Matched Intervention for Healthy Dietary Intake Among Office Workers in a Low- to Middle-Income Country: Protocol for a Cluster Randomized Trial
Source: JMIR Res Protoc. 2025 Sep 30;14:e70293. doi: 10.2196/70293 (PMC12521855; doi:10.2196/70293)
Supplement: Multimedia Appendix 7 [file resprot_v14i1e70293_app7.pdf]

සෞඛ්‍යමත් ආහාර රටාවක් සඳහා මැදිහත්වීම - 03

කන්නෙ මොනවද?
කොහොමද?

Food based dietary guideline

- ආහාර කාණ්ඩ 06
  - ඒ ඒ කාණ්ඩයෙන් දිනකට ගතයුතු ප්‍රමාණය
  - ඒකක / ප්‍රමාණ (servings)
- ඒකක / ප්‍රමාණ (servings) අර්ථ දැක්වීමක්

- **ධාන්‍ය සහ පිශ්ඨය සහිත ආහාර**
  - ඒකක - 06-11 (06)
- **එළවළු**
  - ඒකක - 03-05 (04)
- **පළතුරු**
  - ඒකක - 02-03 (02)
- **මස්, මාළු, බිත්තර සහ ඇට වර්ග**
  - ඒකක - 03-04 (03)
- **කිරි ආශ්‍රිත නිෂ්පාදන**
  - ඒකක - 01-02 (01)
- **තෙල් සහිත ඇට වර්ග**
  - ඒකක - 02-04 (02)

- සෞඛ්‍යමත් නොවන ආහාර - එපා

ආහාර ඒකක පිළිබඳ මාර්ගෝපදේශය

ධාන්‍ය සහ පිශ්ඨය සහිත ආහාර

බත්, වෙනත් ධාන්‍ය සහ පිශ්ඨය සහිත ආහාර –මි.ලී. 200 පීරිසි කෝප්ප 1ක් = බත් හැදි 1ක්
කිරි බත් – සාමාන්‍ය ප්‍රමාණයේ (අඟල් 4-5) කෑලි 1ක්
ආප්ප – සාමාන්‍ය ප්‍රමාණයේ (අඟල් 4-5) 1ක්
ඉදිආප්ප – සාමාන්‍ය ප්‍රමාණයේ (අඟල් 2-3) 3ක්
පිට්ටු – අඟල් 2-3 ප්‍රමාණයේ කෑලි 1ක්
රොටි – සාමාන්‍ය ප්‍රමාණයේ (අඟල් 5-6) රොටියකින් 1/4 ක්

එළවළු

පිසූ එළවළු – මේස හැදි 3ක් = මි.ලී. 200 පීරිසි කෝප්ප 1/2 ක්
අමු එළවළු – මේස හැදි 6ක් = මි.ලී. 200 පීරිසි කෝප්ප 1 ක්

පළතුරු

සාමාන්‍ය ප්‍රමාණයේ පළතුරු 1ක් / ගස්ලබු, අන්නාසි - ගෙඩියකින් 1/6 ක්
කැපූ පළතුරු - මි.ලී. 200 පීරිසි කෝප්ප 1/2 ක්
පළතුරු යුශ - සාමාන්‍ය ප්‍රමාණයේ වීදුරුවකින් 1/2 ක්

ආහාර ඒකක පිළිබඳ මාර්ගෝපදේශය

මස්, මාළු, බිත්තර සහ ඇට වර්ග

මස් / මාළු - සාමාන්‍ය ප්‍රමාණයේ කෑලි 1ක් (ග්‍රෑම් 30ක්)
කරවල - සාමාන්‍ය ප්‍රමාණයේ කෑලි 4-5ක් (ග්‍රෑම් 30ක්)
බිත්තර - 1ක්
තැම්බූ හෝ පිසූ ඇට වර්ග (කඩල,කව්පි,මුං ආදිය) - මේස හැඳි 3ක්

කිරි ආශ්‍රිත නිෂ්පාදන

දියර කිරි - මි.ලී. 200 පීරිසි කෝප්ප 1 ක්
යෝගට් / මිදුන කිරි - මි.ලී. 100 යෝගට් කෝප්ප 1 ක් = මේස හැඳි 3ක්
පිටි කිරි - මේස හැඳි 2ක් (මි.ලී. 200 පීරිසි කෝප්ප 1 කට)

තෙල් සහිත ඇට වර්ග

ඇට වර්ග (රටකජු, ආමන්ඩ් ආදිය) - මේස හැඳි 1ක්

සෞඛ්‍යමත් නොවන ආහාර

බිස්කට්, කේක්, ගැඹුරු තෙලේ බදින ලද ආහාර වලින් 1ක්
සීනි යෙදූ පානයන් - සාමාන්‍ය ප්‍රමාණයේ වීදුරුවකින් 1/2 ක් = මි.ලී. 200ක්

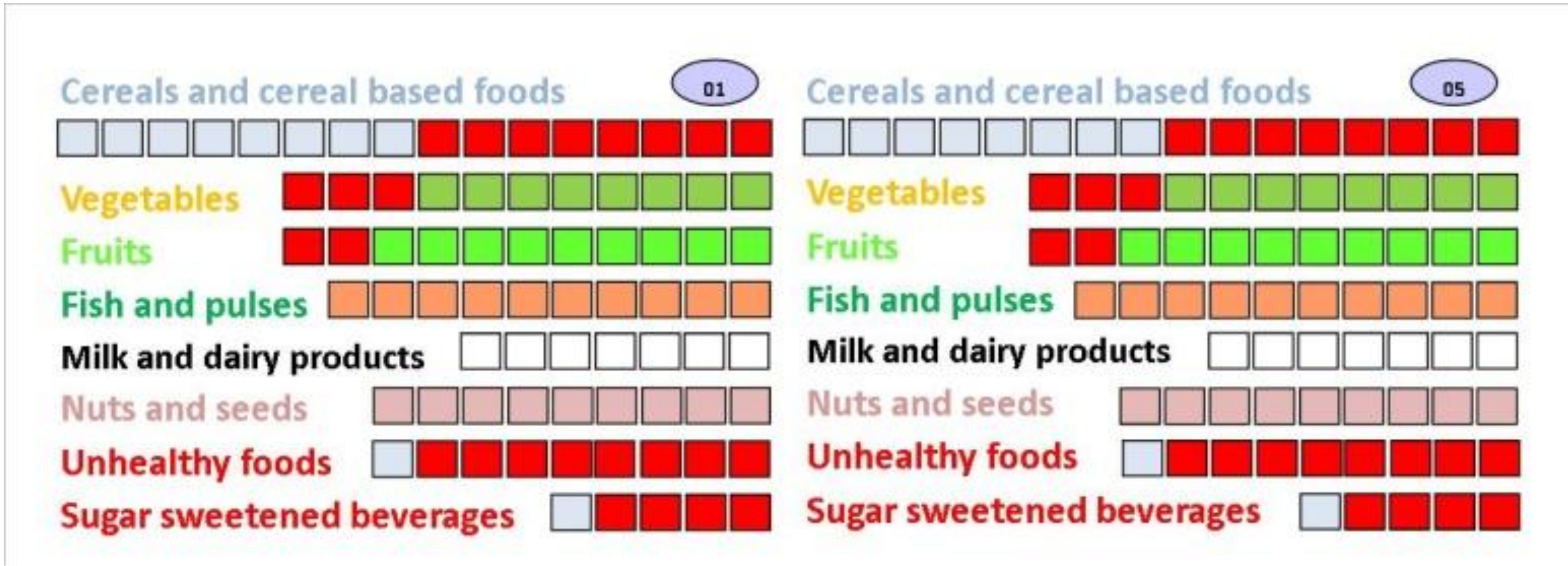
Cereals and cereal based foods
01
Vegetables
Fruits
Fish and pulses
Milk and dairy products
Nuts and seeds
Unhealthy foods
Sugar sweetened beverages
Cereals and cereal based foods
05
Vegetables
Fruits
Fish and pulses
Milk and dairy products
Nuts and seeds
Unhealthy foods
Sugar sweetened beverages

සෞඛ්‍යමත් ජීවිතයක්
