# Supplementary material for: A Stage of Change Theory–Based, Stage-Matched Intervention for Healthy Dietary Intake Among Office Workers in a Low- to Middle-Income Country: Protocol for a Cluster Randomized Trial
Source: JMIR Res Protoc. 2025 Sep 30;14:e70293. doi: 10.2196/70293 (PMC12521855; doi:10.2196/70293)
Supplement: Multimedia Appendix 8 [file resprot_v14i1e70293_app8.pdf]

01

Cereals and cereal based foods

Vegetables

Fruits

Fish and pulses

Milk and dairy products

Nuts and seeds

Unhealthy foods

Sugar sweetened beverages

02

Cereals and cereal based foods

Vegetables

Fruits

Fish and pulses

Milk and dairy products

Nuts and seeds

Unhealthy foods

Sugar sweetened beverages

03

Cereals and cereal based foods

Vegetables

Fruits

Fish and pulses

Milk and dairy products

Nuts and seeds

Unhealthy foods

Sugar sweetened beverages

04

Cereals and cereal based foods

Vegetables

Fruits

Fish and pulses

Milk and dairy products

Nuts and seeds

Unhealthy foods

Sugar sweetened beverages

05

Cereals and cereal based foods

Vegetables

Fruits

Fish and pulses

Milk and dairy products

Nuts and seeds

Unhealthy foods

Sugar sweetened beverages

06

Cereals and cereal based foods

Vegetables

Fruits

Fish and pulses

Milk and dairy products

Nuts and seeds

Unhealthy foods

Sugar sweetened beverages

07

Cereals and cereal based foods

Vegetables

Fruits

Fish and pulses

Milk and dairy products

Nuts and seeds

Unhealthy foods

Sugar sweetened beverages

ආහාර ඒකක පිළිබඳ මාර්ගෝපදේශය

Cereals and cereal based foods

බත්, වෙනත් ධාන්‍ය සහ පිශ්ඨය සහිත ආහාර –
මි.ලී. 200 පීරිසි කෝප්ප 1ක් = බත් හැඳි 1ක්

කිරි බත් – සාමාන්‍ය ප්‍රමාණයේ (අඟල් 4-5) කෑලි 1ක්

ආප්ප – සාමාන්‍ය ප්‍රමාණයේ (අඟල් 4-5) 1ක්

ඉදිආප්ප – සාමාන්‍ය ප්‍රමාණයේ (අඟල් 2-3) 3ක්

පිට්ටු – අඟල් 2-3 ප්‍රමාණයේ කෑලි 1ක්

රොටි – සාමාන්‍ය ප්‍රමාණයේ (අඟල් 5-6) රොටියකින් 1/4 ක්

Vegetables

පිසූ එළවළු – මේස හැඳි 3ක් = මි.ලී. 200 පීරිසි කෝප්ප 1/2 ක්

අමු එළවළු – මේස හැඳි 6ක් = මි.ලී. 200 පීරිසි කෝප්ප 1 ක්

Fruits

සාමාන්‍ය ප්‍රමාණයේ පළතුරු 1ක් / ගස්ලබු, අන්නාසි - ගෙඩියකින් 1/8 ක්

කැපූ පළතුරු - මි.ලී. 200 පීරිසි කෝප්ප 1/2 ක්

පළතුරු යුශ - සාමාන්‍ය ප්‍රමාණයේ වීදුරුවකින් 1/2 ක්

Fish and pulses

මස් / මාළු - සාමාන්‍ය ප්‍රමාණයේ කෑලි 1ක් (ග්‍රෑම් 30ක්)

කරවල - සාමාන්‍ය ප්‍රමාණයේ කෑලි 4-5ක් (ග්‍රෑම් 30ක්)

බිත්තර - 1ක්

තැම්බූ හෝ පිසූ ඇට වර්ග (කඩල,කව්පි,මුං ආදිය) - මේස හැඳි 3ක්

Milk and dairy products

දියර කිරි - මි.ලී. 200 පීරිසි කෝප්ප 1 ක්

යෝගට් / මිදුන කිරි - මි.ලී. 100 යෝගට් කෝප්ප 1 ක් = මේස හැඳි 3ක්

පිටි කිරි - මේස හැඳි 2ක් (මි.ලී. 200 පීරිසි කෝප්ප 1 කට)

ඇට වර්ග (රටකජු, ආමන්ඩ් ආදිය) - මේස හැඳි 1ක්

Unhealthy foods

බිස්කට්, කේක්, ගැඹුරු තෙලේ බදින ලද ආහාර වලින් 1ක්

සීනි යෙදූ පානයන් - සාමාන්‍ය ප්‍රමාණයේ වීදුරුවකින් 1/2 ක් = මි.ලී. 200ක්

(ඉහත දැක්වෙන ආහාර ප්‍රමාණයන් එක් ඒකකයක් ලෙස සලකන්න)

බත් හැඳි 1ක් = ලොකු පොල්කටු හැඳි 1 = කුඩා පොල්කටු හැඳි 2 = මේස හැඳි 4

සාමාන්‍ය ප්‍රමාණයේ පොල්කටු හැඳි 1ක් = මේස හැඳි 3

කුඩා පොල්කටු හැඳි 1 = මේස හැඳි 2

මි.ලී. 200 පීරිසි කෝප්ප 1 ක් = මේස හැඳි 6
